# Seasonal structural stability promoted by forest diversity and composition explains overyielding

**DOI:** 10.1002/ecy.70055

**Published:** 2025-03-17

**Authors:** J. Antonio Guzmán Q., Maria H. Park, Laura J. Williams, Jeannine Cavender‐Bares

**Affiliations:** ^1^ Department of Ecology, Evolution, and Behavior University of Minnesota Saint Paul Minnesota USA; ^2^ Department of Organismic and Evolutionary Biology Harvard University Cambridge Massachusetts USA; ^3^ Hawkesbury Institute for the Environment Western Sydney University Penrith New South Wales Australia

**Keywords:** biodiversity, forest stability, net biodiversity effect, phenology, species variability, UAV‐LiDAR

## Abstract

The stability of forest productivity is a widely studied phenomenon often associated with tree species diversity. Yet, drivers of stability in forest structure and its consequences for forest productivity remain poorly understood. Using a large (10 ha) young tree diversity experiment, we evaluated how forest structure and multiple dimensions of diversity and composition are related to remotely sensed structural metrics and their stability through the growing season. We then examined whether structural stability (SS) across the growing season (April–October) could explain overyielding (i.e., the net biodiversity effect, NBE) in annual wood productivity. Using Uncrewed Aerial Vehicle‐Light Detecting and Ranging (UAV‐LiDAR), we surveyed experimental tree communities eight times at regular intervals from before bud break to after leaf senescence to derive metrics associated with canopy height heterogeneity, fractional plant cover, and forest structural complexity (based on fractal geometry). The inverse coefficients of variation for each of these three metrics through the season were used as measures of SS. These metrics were then coupled with annual tree inventories to evaluate their relationships with the NBE. Our findings indicate that wood volume and, to some extent, multiple dimensions of diversity and composition (i.e., taxonomic, phylogenetic, and functional) explain remotely sensed metrics of forest structure and their SS. Increases in wood volume as well as functional and phylogenetic diversity and variability (a measure of diversity independent of species richness) were linked to higher SS of forest complexity and canopy height heterogeneity. We further found that higher SS of forest complexity and fractional plant cover were associated with increased overyielding, which was mostly attributable to the complementarity effect. Structural equation models indicate that the stability of structural complexity explains more variation in NBE among plots than dimensions of diversity or variability, highlighting its value as an informative metric that likely integrates multiple drivers associated with overyielding. This study highlights the potential to integrate remote sensing and ecology to disentangle the role of forest SS in shaping ecological processes.

## INTRODUCTION

Diverse tree communities are critical for the maintenance of healthy and resilient forest ecosystems (Messier et al., 2022). Diverse communities are known to enhance ecosystem functions, including nutrient cycling, water use, soil microbial processes, and primary productivity (Hooper et al., 2005; Kunert et al., 2012). Furthermore, increases in productivity and stability are often attributed to tree diversity (Dolezal et al., 2024). One purported driver of increased productivity in diverse tree communities is the spatial complementarity of tree crowns. As tree species with diverse architectures fill different portions of the available space, they minimize overlap among trees and reduce the amount of light that reaches the forest floor (Juchheim et al., 2019; Seidel et al., 2013; Williams et al., 2017, 2021). Forest stability—measured as the temporal variation of forest attributes—has been proposed to be an emergent property of diversity. The stability‐diversity relationship is explained by the insurance hypothesis, where diverse communities provide more guarantees that some species will maintain forest function even if others fail (Loreau et al., 2021; Yachi & Loreau, 1999). Although diversity and stability are essential to forest ecosystem function, they are difficult to monitor using traditional forest inventories. Developing tools to remotely sense forest diversity and its stability is critical to advancing our understanding of managing biodiversity on land (Cavender‐Bares et al., 2022).

Recent evidence from tree biodiversity experiments indicates that increases in species richness can lead to stable forest communities via species‐asynchrony—temporal differences in growth patterns among species—given that interannual fluctuations in growth can buffer community productivity against species‐specific stress‐related productivity declines (Schnabel et al., 2019, 2021). This phenomenon has also been shown in natural mixtures where species‐asynchrony can counterbalance the negative climate‐related effects on the stability of forest productivity (del Río et al., 2022). Most of the current body of knowledge about forest stability comes from studies of the long‐term effects of tree diversity. Although short‐term effects (e.g., across seasons within a year) of diversity in stability have received less attention, forest stability can be important in a single growing season given that species differ in their intra‐annual timing of development and growth. From this perspective, species‐asynchrony within a growing season may result from species‐specific phenological rhythms (e.g., differences in the timing of leaf flushing or senescence) and responses to environmental fluctuations that are likely to influence forest structure (Smith et al., 2019). Consequently, diverse forest communities that integrate different growth and resource acquisition strategies across time are anticipated to sustain high levels of SS throughout the growing season. SS, in turn, may help these communities maintain high levels of productivity. However, seasonal fluctuations in forest structure are difficult to assess using traditional forest inventory methods, which rarely capture forest structure in three dimensions.

Remote sensing technologies are advancing our ability to frequently measure and monitor forest structure, diversity, and composition (Turner, 2014). Specifically, advances in Light Detecting and Ranging (LiDAR) are revolutionizing how we measure forest structure and structural diversity (Atkins et al. 2023a). Forest taxonomic diversity, particularly species richness, has long been associated with LiDAR structural metrics such as canopy height (Robinson et al., 2018), *HH*_CV_ (Torresani et al., 2020), and structural complexity (Ehbrecht et al., 2017). Structural metrics derived from LiDAR have also been used to evaluate mechanisms associated with the net biodiversity effects (NBEs) on productivity (i.e., overyielding) such as crown complementarity (Kunz et al., 2019) and forest complexity (Ray et al., 2023). Despite these advances, it remains unclear how structural attributes of forests measured by LiDAR can be coupled to multiple dimensions of diversity and composition (e.g., phylogenetic or functional). Moreover, the assessment of SS across the growing season and its association with forest diversity, composition, and productivity is not well understood.

**FIGURE 1:**
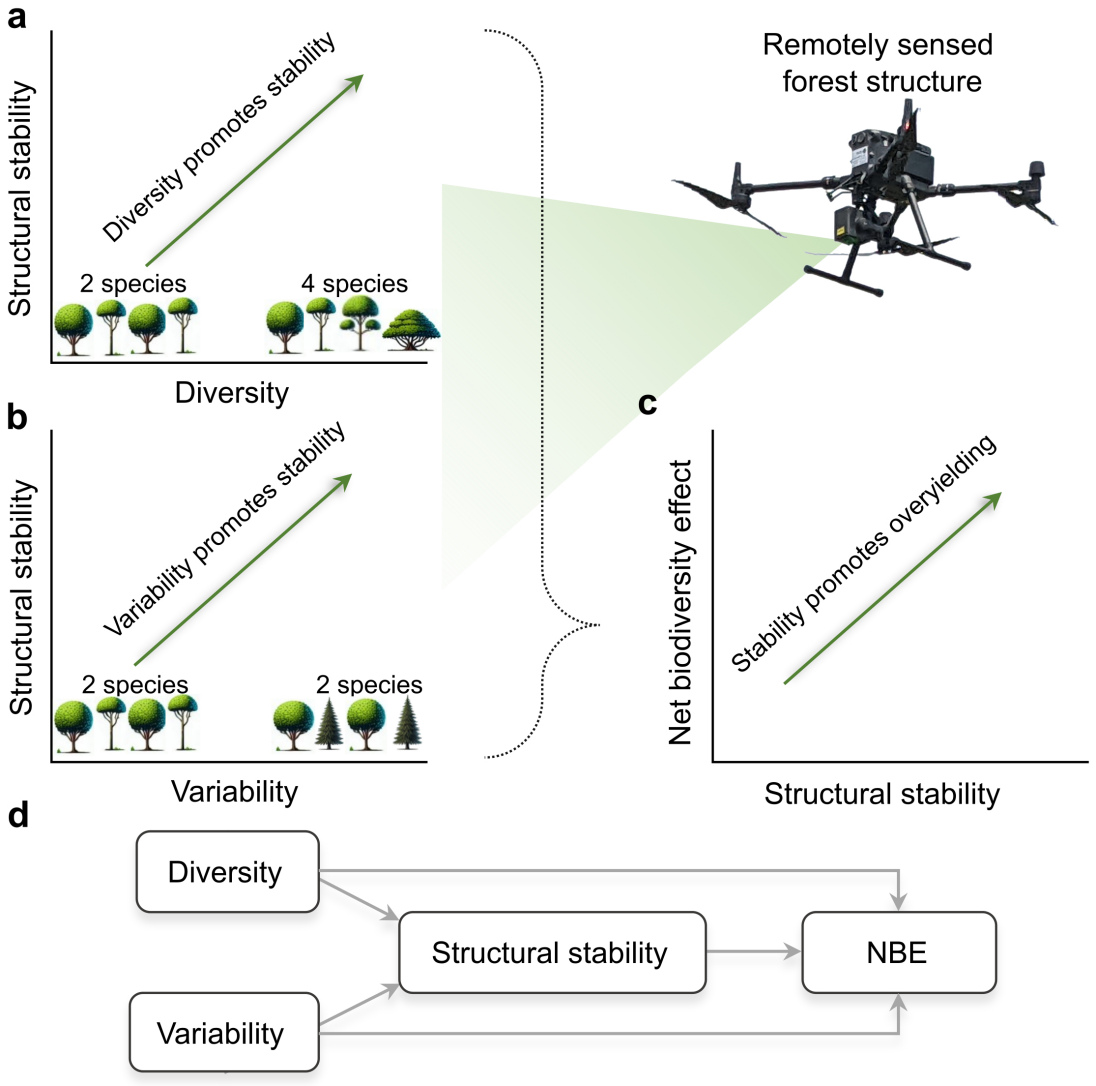
Schematic representation of how remotely sensed structural stability (SS) through the growing season is promoted by multiple dimensions (i.e., taxonomic, phylogenetic, or functional) of diversity and variability which, in turn, explain net biodiversity (NBE) effects of productivity (i.e., overyielding). (a) Expected trends with increases in diversity where trees resample species. (b) Expected trends with variability**—**the degree to which species in a community are taxonomically, phylogenetically, or functionally different—where contrasting tree shapes and colors increases the community variability regardless of the number of species. (c) Expected trends of NBE indirectly promoted by diversity and variability. (d) Integration and comparison of our hypothesis using structural equation models.

Here we aim to assess (1) how experimentally controlled variation in multiple dimensions of forest diversity and composition is linked to forest structural metrics derived from LiDAR onboard an Uncrewed Aerial Vehicle (UAV), and (2) how changes in forest structure throughout the growing season (i.e., SS) contribute to NBEs on forest productivity. We focus on three forest structural metrics derived from LiDAR that collectively capture vertical and horizontal dimensions: HH_CV_ as a descriptor of height variation (Torresani et al., 2020, 2023), fractional plant cover (FC) as a descriptor of light attenuation and vegetation density, and a fractal dimension (*d*_D_) metric that captures the three‐dimensional arrangements of tree structure (i.e., forest structural complexity) (Guzmán et al., 2020). The inverse of the coefficient of variation of these metrics throughout the growing season was used as descriptors of SS. We used the *Forest and Biodiversity* (FAB2) experiment (Cavender‐Bares et al., 2024) to test a series of hypotheses about the interrelationships of forest diversity, structure, and stability, controlling for forest age, tree density, climate, and management. We test three specific hypotheses: First, increases in the volume occupied by a forest assemblage, measured on the ground as plot wood volume, are associated with LiDAR‐derived metrics of structure and their stability. Second, taxonomic, phylogenetic, and functional measures of diversity (which covary with species richness) and variability—the degree to which species in a community are taxonomically, evolutionarily, or functionally different regardless of species richness (Helmus et al., 2007)—are likely to explain LiDAR‐derived metrics of forest structure and stability (Figure 1a,b). Here we further posit that variability metrics will explain structural complexity and stability better than diversity metrics. In other words, assemblages with functionally or phylogenetically different species, regardless of species richness level, are prone to include a diverse range of tree architectures, phenological rhythms, and resource capture strategies that influence structure and stability. Finally, we hypothesize that diversity and variability contribute to SS, resulting in increases in NBE on forest productivity (Figure 1c). Here we posit that greater differences among interacting trees will result in crown complementary and phenological asynchrony, giving rise to SS and increased resource capture across the season. We test these hypotheses individually and examine their relative importance using structural equation models (SEMs) (Figure 1d).

## METHODS AND MATERIALS

### Study site

We conducted this study in the Forest Biodiversity Experiment 2 (FAB2) located at the Cedar Creek Ecosystem Science Reserve (CCESR), Minnesota, USA (Cavender‐Bares et al., 2024) (45° 24′ 22.6″ N and 93° 11′ 30.0″ W). This site is characterized by a flat outwash plain with infertile, excessively drained soils consisting of upward of 90% sand as part of the Anoka Sand Plain. This reserve has a humid continental climate with warm summers and cold winters. The region experiences a mean annual temperature close to 7°C with 660 mm of mean annual precipitation, and the growing season generally extends from late April to early October (Appendix S1: Figure S1). FAB2 was designed to evaluate the influence of multiple dimensions of diversity and composition on long‐term community processes and ecosystem function and resilience. FAB2 is a tree diversity experiment established in 2016–2017 in an abandoned old‐field previously dominated by herbaceous species and fenced to exclude large mammalian herbivores. The experiment covers approximately 6.5 ha and has trees planted 1 m apart in 148 small plots (100 m^2^) and 30 large (400 m^2^) plots that are randomly arranged spatially in three blocks. Twelve native species were planted in replicated monocultures, and in polycultures of two‐, four‐, six‐, and twelve‐species each spanning a range of functional and phylogenetic variability (for details, see Cavender‐Bares et al., 2024). These species represent eight deciduous angiosperms (i.e., *Acer negundo*, *A. rubrum*, *Betula papyrifera*, *Quercus alba*, *Q. ellipsoidalis*, *Q. macrocarpa*, *Q. rubra*, and *Tilia americana*) and four evergreen gymnosperms (i.e., *Juniperus virginiana*, *Pinus banksiana*, *P. resinosa*, and *P. strobus*). For this study, we divided the 400 m^2^ (20 m x 20 m) plots into four quadrants, resulting in four 100 m^2^ (10 m × 10 m) plots to increase the number of replicates and to standardize plot size. The height and diameter (at the root collar and at breast height for trees >1.3 m in height) of each tree in the experiment has been measured each year at the end of the growing season.

### Tree wood volume, productivity, and NBEs

Using the annual inventories of tree diameter and height that coincided with the study period (i.e., 2021 and 2022), we estimated wood volume for each tree, plot‐level wood volume, annual tree and plot wood productivity, and the NBEs (sensu Loreau & Hector, 2021, also referred to as overyielding) on annual wood productivity. Specifically, we estimated tree wood volume (m^3^) using the geometric formula for conoid and conoidoid volume (CCV) and/or their joint sums. For trees shorter than 1.3 m, we used the conoid volume (CV):

(1) $$\mathrm{CV}=\frac{H\times \pi \times D_{\mathrm{rc}}^{2}}{12},$$

where *H* is the height of the tree from the root collar to the apical meristem, and *D* is the stem diameter at the root collar (rc). For trees greater than 1.3 m, we used the combined CCV formula as

(2) $$\mathrm{CVV}=\left[H_{\mathrm{DBH}}\times \left(D_{\mathrm{rc}}^{2}+\mathrm{DBH}\times D_{\mathrm{rc}}+{\mathrm{DBH}}^{2}\right)\times \frac{\pi }{12}\right]+\left(\frac{H^{\prime }\times \pi \times {\mathrm{DBH}}^{2}}{12}\right),$$

where *H*_DBH_ is height at 1.3 m, DBH is diameter at breast height, and *H′* is the additional height above 1.3 m to the apical meristem. We removed the edge row and column of planted trees from each plot, eliminating the outer 0.5 m of the perimeter to avoid the edge effect, providing an effective plot area of 81 m^2^. Annual tree wood productivity per hectare (AWP_tree_, m^3^ year^−1^ ha^−1^) was then estimated as the difference in tree volume between the 2022 and 2021 inventories divided by the approximate time interval between inventories, with units reported in years (i.e., 365 days/year) and the area of the plot (i.e., 0.0081 ha/plot). Plot wood volume (m^3^ ha^−1^) and annual wood productivity (AWP_plot_, m^3^ year^−1^ ha^−1^) per hectare were estimated as the sum of tree volume and AWP_tree_. Using the AWP_tree_ estimations, we estimate NBE on productivity following Loreau and Hector (2001) as

(3) $$\mathrm{NBE}={\mathrm{AWP}}_{\text{plot}}-{\mathrm{AWP}}_{\text{expected}},$$

where AWP_expected_ is the expected annual plot productivity calculated as

(4) $${\mathrm{AWP}}_{\text{expected}}=\sum \left({\mathrm{AWP}}_{i}\times p_{i}\right),$$

where AWP is the mean AWP_plot_ of the species *i* from monocultures that were planted before 2019, and *p* is the proportion of trees of the species *i* planted in the mixture. Our NBE estimate here includes tree mortality as part of the effect of diversity but excludes plots with trees replanted in 2022 to avoid potential increases in biomass due to the addition of trees.

### Diversity metrics

For each plot, we estimated multiple dimensions of diversity associated with taxonomic diversity (i.e., species richness), phylogenetic diversity (PD), and functional diversity (FD) though Hill numbers following Chao et al. (2014). Diversity metrics were all calculated such that they increase with the number of species. Species richness was estimated as the number of species in the plot after tree mortality. PD was estimated as the total branch length of a pruned phylogenetic tree derived from Smith and Brown (2018). Finally, FD was estimated as the effective total functional distance between species using functional traits related to growth, structure, and light strategies or requirements (i.e., wood density, leaf mass area, maximum tree height, relative growth rate, and shade tolerance) (Appendix S1: Table S1). We computed these metrics using the “*hill_taxa*,” “*hill_phylo*,” and “*hill_func*” functions of the *hillR* package (Li, 2018) of R (R Core Team, 2023) with a Hill order *q =* 0. The phylogenetic tree was accessed through the “*phylo.maker*” function of the *V.PhyloMaker2* package (Jin & Qian, 2022) of R using the third scenario.

### Taxonomic, phylogenetic, and functional variability

We used the phylogenetic species variability metric proposed by Helmus et al. (2007)—herein referred to as variability—to quantify the degree of dissimilarity among species within communities as a function of their taxonomic, phylogenetic, or functional distances. This metric, which is explicitly designed to be independent of species richness, is computed on phylogenetic trees or dendrograms and provides a range of values between 0 (e.g., high relatedness or similarity among species) to 1 (e.g., low relatedness or similarity among species). The metric describes the degree of difference among members of a community, regardless of how many species are included. Similar to PD above, we used a pruned phylogenetic tree derived from Smith and Brown (2018) to estimate phylogenetic variability. We calculated functional variability following the approach used in the design of the experiment in which a dendrogram constructed from a multivariate Euclidean distance matrix of functional traits (Appendix S1: Table S1) is forced into the format of a phylogenetic tree using the “*as.phylo*” command in the *ape* package (Paradis et al., 2004). The resulting functional tree was used then to calculate variability. Finally, we derived taxonomic variability by using a hierarchical clustering tree of taxonomic distinctness (Clarke & Warwick, 1998) considering order, family, genus, and species. The taxonomic distinctness was estimated using the “*taxa2dist*” function from the *vegan* package (Oksanen et al., 2022). The clustering tree of taxonomic distinctness resembles the pruned phylogenetic tree (Appendix S1: Figure S1) but differs from it in that branch length distances are each a single step and not informed by time‐calibrated molecular distances among taxa. We excluded monocultures from our analyses of variability, given that the metric is undefined for monocultures in the absence of measured intraspecific variability. This variability metric was estimated using the function “*psv*” from the *picante* package (Kembel et al., 2010).

### LiDAR data collection and processing

We collected LiDAR data across the FAB experiment eight times during 2022 on the following days of the year: 100, 138, 163, 188, 215, 250, 261, and 297. Flights were conducted throughout the growing season, spanning the entire favorable period for growth based on cumulative growing degree days (Appendix S1: Figure S2), starting prior to leaf out and extending until after leaf senescence for the deciduous species. Data were collected using a DJI Zenmuse L1 sensor onboard an Uncrewed Aerial Vehicle (UAV) DJI Matrice 300. This sensor integrates a Livox LiDAR module, an inertial measurement unit, and an RGB camera on a 3‐axis stabilized gimbal. The LiDAR module has a conic footprint on the ground with a field of view of 77.2° vertical and 70.4° horizontal, enabling it to capture up to three returns for each pulse. All the surveys were conducted using autonomous flights programmed to take place at a speed of 8 m/s at 50 m above the ground. These surveys were done in an area of ~5.8 ha with 85% overlap between sidetracks, allowing capture of dense point clouds (~2100 points/m^2^). During these flights, we also collected static GNSS data using an Emlid Reach RS2+ receiver to enable kinematic corrections or postprocessing corrections in case of signal loss. The data collected from the LiDAR sensor and GNSS receiver were processed in DJI Terra, delivering true color point clouds using the optimization option for manufacture strip alignment. The resulting point clouds were then processed through BayesStripAlign 2.24 to ensure a proper alignment among flight lines.

We treated the resulting point clouds in a series of steps to ensure data quality. First, points were classified as ground and non‐ground and then normalized by height (Appendix S1). These procedures were done using “*lasthin*,” “*lasground*,” and “*lasheight*” of *LAStools* (Isenburg, 2014). Then, we used a multi‐polygon layer of the spatial limits of the experiment (e.g., figure 2 in Cavender‐Bares et al., 2024) with a buffer of −0.5 m from the edge to segment point clouds. This buffer was applied to exclude trees planted at the edge, similar to a previous section. We aligned the multi‐polygon layers within each survey using six long‐term ground control points along the experiment to ensure spatial consistency between geospatial products. The segmented point clouds were then filtered by noise using the Statistical Outliers Removal (SOR) algorithm, considering 30 neighboring points and a threshold value equal to the 97.5% quantile. The filtering of noisy points was applied just on points above 0.25 m from the ground, considered as vegetation. Once the segmented point clouds were filtered, we decimated them further to reduce the redundancy of points by selecting a point within a voxel grid of 1 cm of resolution. The filtering of points by noise and decimation procedure were done using the *lidR* package (Roussel et al., 2020).

### LiDAR‐derived metrics

We derived three metrics to characterize the canopy heterogeneity, cover, and complexity from point clouds of each plot. Overall, we estimated: (i) the coefficient of variation of canopy height (HH_cv_), (ii) the fractional cover (FC), and (iii) the fractal dimension (*d*_D_), which integrate the HH_CV_, the light attenuation associated with plant density, and three‐dimensional arrangements of plant structures, respectively. We estimated these metrics only on plots with a canopy height higher than 1.5 m at the eight times that were surveyed, and on plots where most trees were planted before 2019 (i.e., mean planted year per plot) (*n* = 166). Specifically, we computed *HH*_cv_ by estimating the coefficient of variation from cells of Digital Canopy Models from the plots with a 0.1 m resolution. Likewise, we estimated FC following (Boucher et al., 2023) by dividing the sum of the inverse of return number (i.e., 1/return number) above the ground (i.e., > 0.25 m above the ground) by the total number of pulses from the plot. Finally, we derived the *d*_D_ through the box‐counting method as presented in Guzmán et al. (2020), but computed using Hill diversity (Jost, 2006). As such, a point cloud from a plot can be covered using a voxel (*N*_1_ = 1) of edge size *S*_1_ (m), but as the voxel edge size (*S*) is reduced (*S*_1_ > *S*_2_ > *S*_*n*_), the number of voxels required (*N* > 1) to cover it increases. *N* increases as a power function; thus, their slope (*d*) and intercept (*a*) can be computed using a linear model, as follows:

(5) $$\log\left(N\right)=d\times \log\left(\frac{1}{S}\right)+a.$$

In order to incorporate Hill diversity into this formulation, *N* is replaced by estimating the Hill diversity (*D*) at each scale (i.e., *S*) as follows:

(6) $$q_{D}={\left(\sum_{i=1}^{S}p_{i}^{q}\right)}^{\frac{1}{\left(1-q\right)}},$$

where *p*_*i*_ is the proportion of points that fall in a given voxel, and *q* is the different Hill orders (i.e., *q =* 0, *q =* 0.999, *q =* 2). This procedure allows us to compute different levels of fractal dimensions associated with the Hill orders (*d*_D_). In that regard, *d* resembles the box‐counting dimension when *q* = 0, *d* resembles the information dimension when *q* approximates 1, and *d* resembles the correlation dimension when *q* = 2. Here, we focused our attention on when *q* approximates 1 as it may help to reduce the differences in point density among plots (Liu et al., 2022). We solved the previous linear model by applying Standardized Major Axis (SMA) regressions using the “*sma*” function of the *smart* package in R (Warton et al., 2012). To avoid the apparent nonlinear trends due to occlusion effects on the point clouds, we used a sequence of voxel edge sizes (*S*) between 0.1 and 2.25 m of edge length. The estimations of *d*_D_ through voxelization were done with the help of the *lidR* (Roussel et al., 2020) and *rTLS* packages (Guzmán et al., 2021).

### Seasonal SS

We used the inverse of the coefficient of variation as a descriptor of the magnitude of seasonal fluctuation in forest structure, and thus its SS across the season. This descriptor was estimated for each LiDAR metric (i.e., HH_cv_, FC, and *d*_D_) as follows:

(7) $$\mathrm{SS}=\frac{\underline{x}}{\sigma },$$

where σ is the standard deviation and *x* is the mean of each metric from the eight surveys during the growing season. A higher value of SS describes a plot where its canopy height heterogeneity (SS_HHcv_), fractional plant cover (SS_FC_), or structural complexity (SS_*d*D_) is stable across the growing season.

### Data analysis

First, we performed separate linear mixed models to evaluate the extent to which plot wood volume, diversity, and variability explain changes in LiDAR‐derived metrics. These linear mixed models also considered the observation period effect (i.e., as a continuous variable) and its interaction with the aforementioned parameters. For these models, we treated plots nested within experimental blocks as a repeated random effect. Second, we performed linear regressions between LiDAR‐derived metrics at different observation periods and plot volume, diversity, and variability to determine how much of the variance (coefficient of determination, *R* ^2^) was explained by changes in structure, diversity, and composition. Third, we applied linear mixed models to test how plot wood volume, diversity, and variability explain the SS of metrics derived from LiDAR. Fourth, we assessed the association of SS with NBE using linear regression models. Finally, we performed SEMs in the form of structural regression with endogenous variables to assess pathways of how both diversity and variability relate to forest SS, and how the latter, in turn, explains NBE (Figure 1d). These SEMs were evaluated using diversity and variability as exogenous variables described by their dimensions (i.e., taxonomic, phylogenetic, and functional), and two endogenous variables of NBE and SS; the former one associated with each LiDAR‐derived metric. Linear mixed models were fitted using the *lme4* package (Bates et al., 2015), while the linear regressions were performed using the *ggpmisc* package (Aphalo, 2023) through *ggplot2* (Wickham, 2016). SEM models were performed using the *lavaan* package in R (Rosseel, 2012). Metrics of plot wood volume, dimensions of diversity, and structural and SS metrics derived from LiDAR were log‐transformed for the analysis to allow residuals to approximate a normal distribution. Some of the linear regressions did not show homogeneity of variance but were kept linear to facilitate the interpretation of the relationships.

## RESULTS

### Relationships between wood volume and structural metrics derived from LiDAR

**FIGURE 2:**
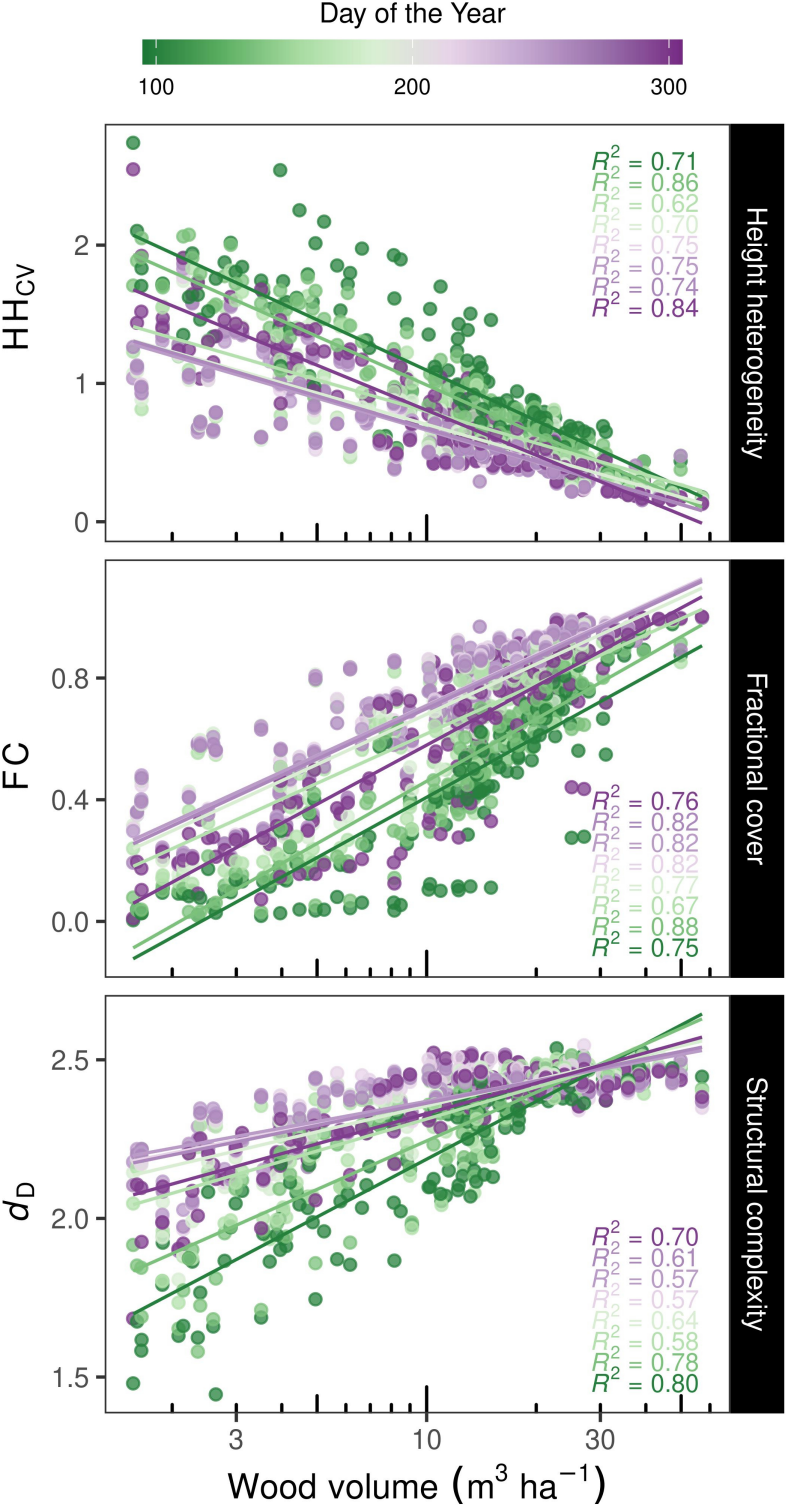
Relationships of plot wood volume with LiDAR‐derived metrics at different observation periods through the growing season. HH_CV_ describes the coefficient of variation of canopy height heterogeneity, FC the fractional plant cover, and *d*_D_ the fractal dimension. Each point represents a plot within an area of 81 m^2^ (see Appendix S1: Figure S3 for the significance of the regression models).

**FIGURE 3:**
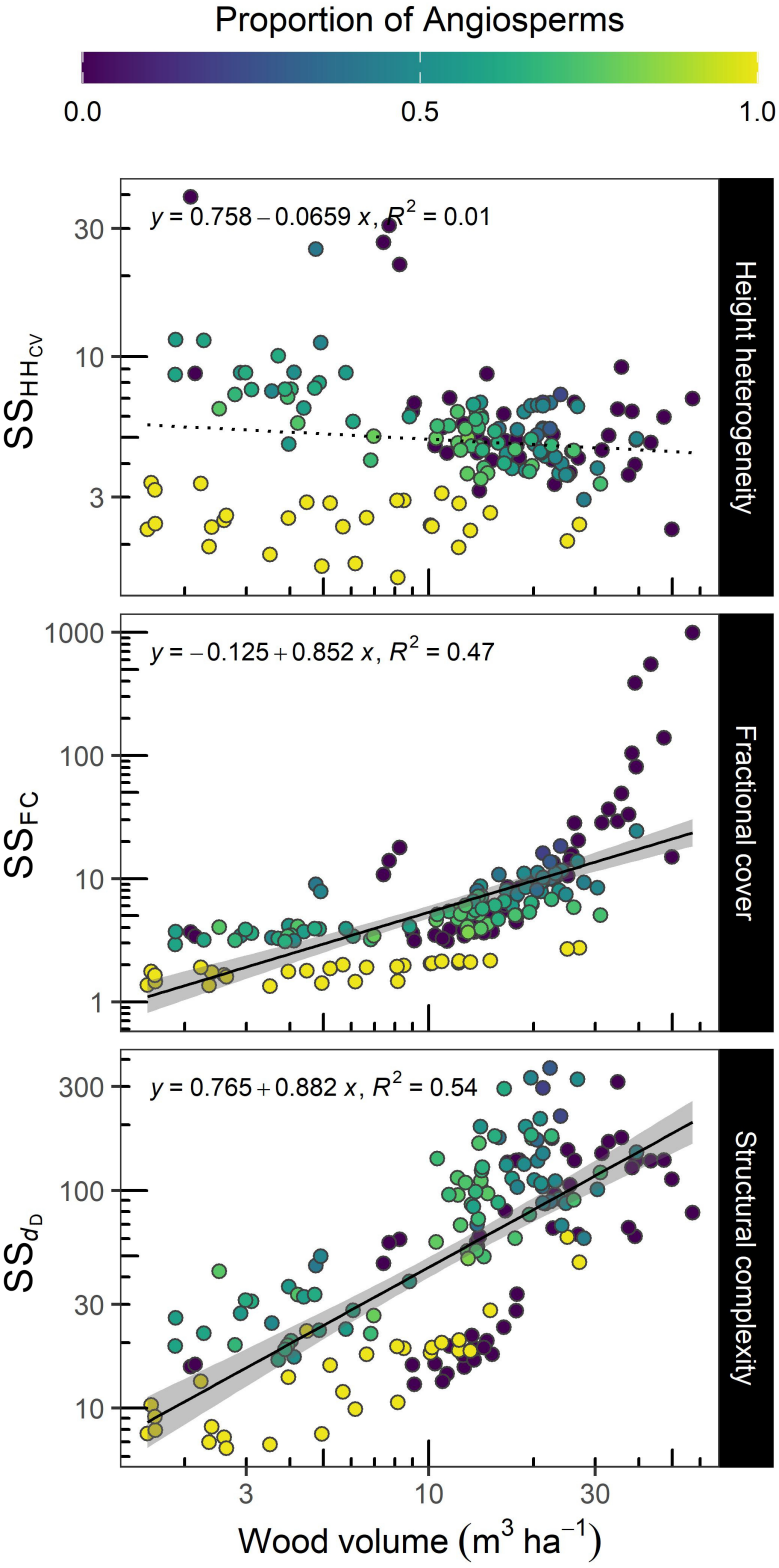
Association of plot wood volume with the structural stability (SS) of LiDAR‐derived metrics through the growing season. HH_CV_ describes the coefficient of variation of canopy height heterogeneity, FC the fractional plant cover, and *d*_D_ the fractal dimension. Colors represent the proportion of angiosperm trees planted in each plot. Each point represents a plot within an area of 81 m^2^.

The relationships between plot wood volume and LiDAR‐derived metrics of forest structure across the growing season revealed that increases in wood volume were strongly associated with reductions in *HH*_CV_ and increases in fractional cover (FC) and structural complexity (*d*_D_) (Figure 2; see Appendix S1: Figure S3 for further details). Differences in trends among observation periods emerged in parallel with seasonal changes, where communities showed reduced HH_CV_ and higher FC and *d*_D_ close to the peak of the growing season (DOY 215 or August 15) Appendix S1: (Figure S4). The importance of seasonal change in forest structure was also clear in the linear mixed models (Appendix S1: Table S2), but models also revealed an interaction between the observation period (i.e., DOY) and wood volume. Moreover, our measures of forest SS across the growing season showed that increases in wood volume explained increases in the stability of fractional plant cover (SS_FC_) and forest complexity (SS_*d*D_), but did not explain the stability of SS_HHcv_ (Figure 3). Furthermore, it appeared that communities composed of gymnosperms and mixtures of angiosperms and gymnosperms presented higher SS_HHcv_, SS_FC_, and SS_*d*D_ than communities composed solely of angiosperms (Appendix S1: Figure S5).

### Association of multiple dimensions of diversity with structural metrics derived from LiDAR

**FIGURE 4:**
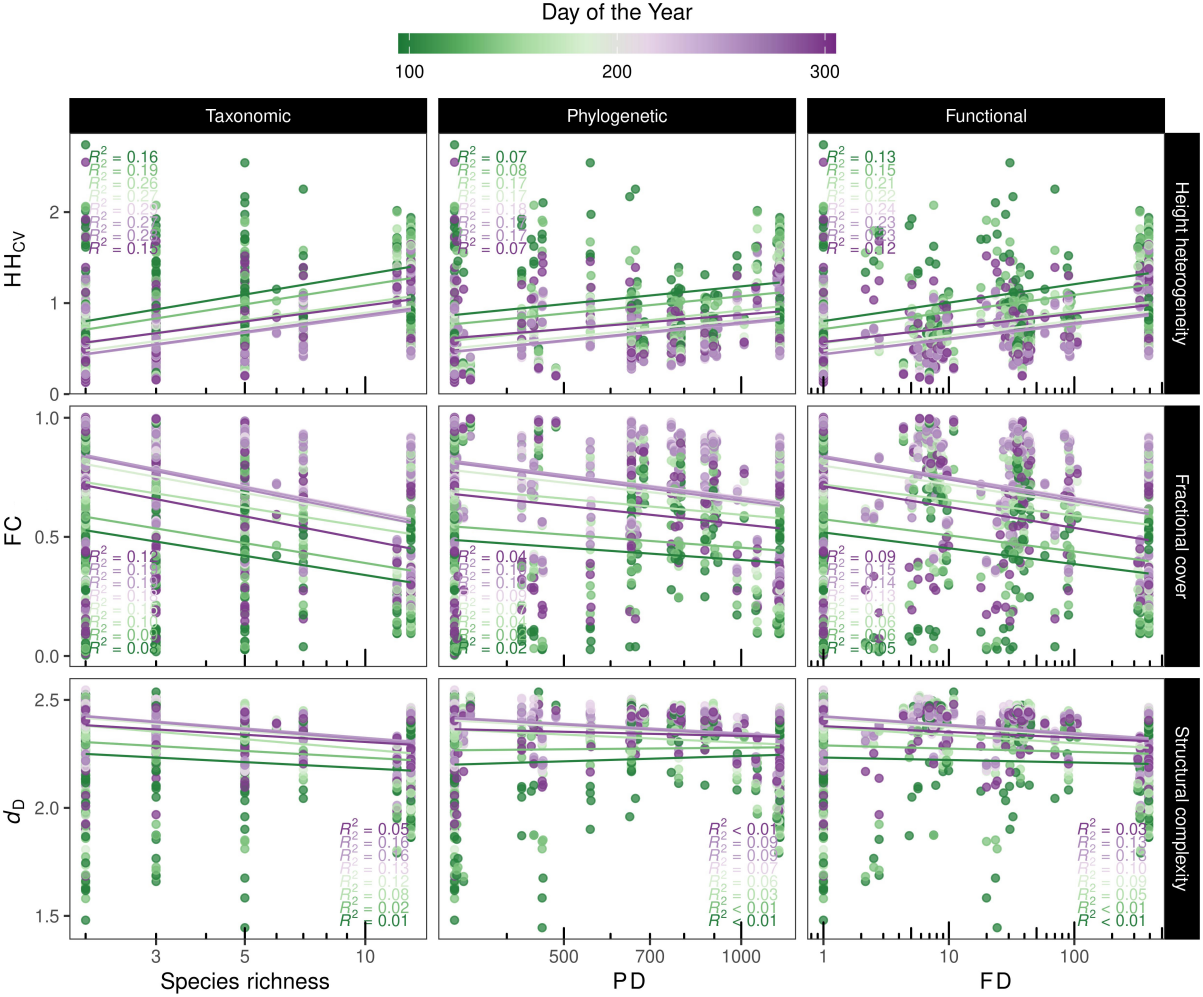
Relationships of multiple dimensions of diversity with LiDAR‐derived metrics at different observation periods during the growing season. HH_CV_ describes the coefficient of variation of canopy height heterogeneity, FC the fractional plant cover, and *d*_D_ the fractal dimension. See Appendix S1: Figure S6 for the significance of the regression models.

**FIGURE 5:**
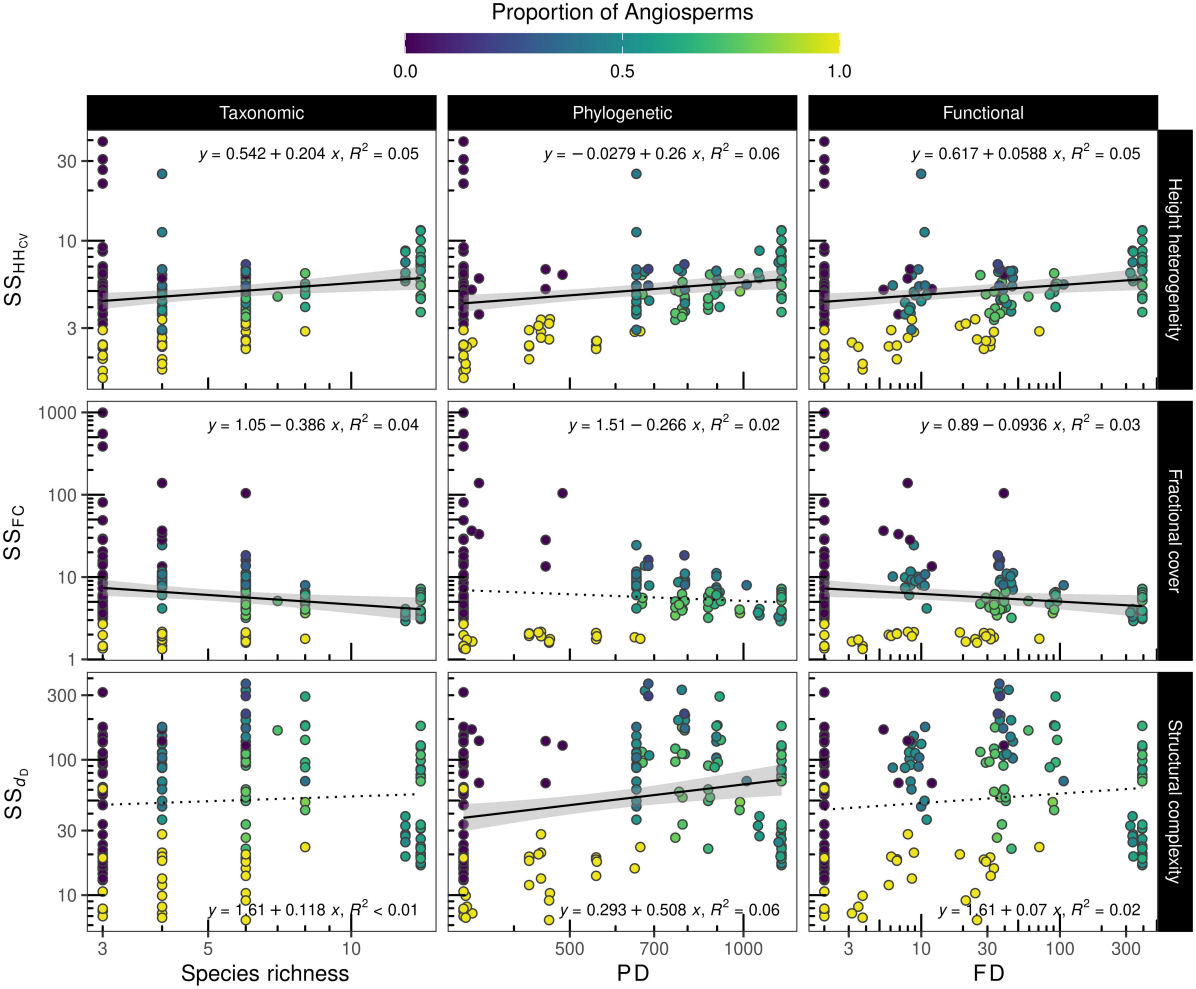
Association of multiple dimensions of diversity with the structural stability (SS) of LiDAR‐derived metrics through the growing season. HH_CV_ describes the coefficient of variation of canopy height heterogeneity, FC the fractional plant cover, and *d*_D_ the fractal dimension. Most of these relationships were not statistically significant. Colors represent the proportion of angiosperm trees that were planted for each plot.

Analyses of the relationships between multiple dimensions of diversity and LiDAR‐derived metrics revealed that increases in species richness, PD (calculated as effective total branch length), and FD (calculated as the effective total functional distance between species) tended to be associated with increases in HH_CV_ and, to some extent, decreases in FC and *d*_D_ (Figure 4; see Appendix S1: Table S3 and Figure S6 with greater detail). In most cases, the variance explained by these relationships was low (i.e., ≤ 29%) but appeared to be better explained in observation periods close to the peak of the growing season. These results were also supported by the linear mixed models (Appendix S1: Table S3), which indicated that observation periods had a significant influence on these relationships. When comparing diversity metrics according to the coefficient of determination, species richness appeared to better explain variations in LiDAR‐derived metrics than phylogenetic or FD. Among the three LiDAR‐derived metrics, HH_CV_ was better explained by variations in diversity than FC and *d*_D_, which were poorly explained (*R* ^2^ < 0.19). By examining the seasonal SS, our results also revealed that increases in diversity were modestly associated with increases in SS_HHcv_ (Figure 5). In addition, increases in SS_FC_ were associated with reductions in species richness and FD, but not PD, while increases in SS_*d*D_ were modestly explained by increases in PD, but not species richness or FD.

### Association of multiple dimensions of variability with structural metrics derived from LiDAR

**FIGURE 6:**
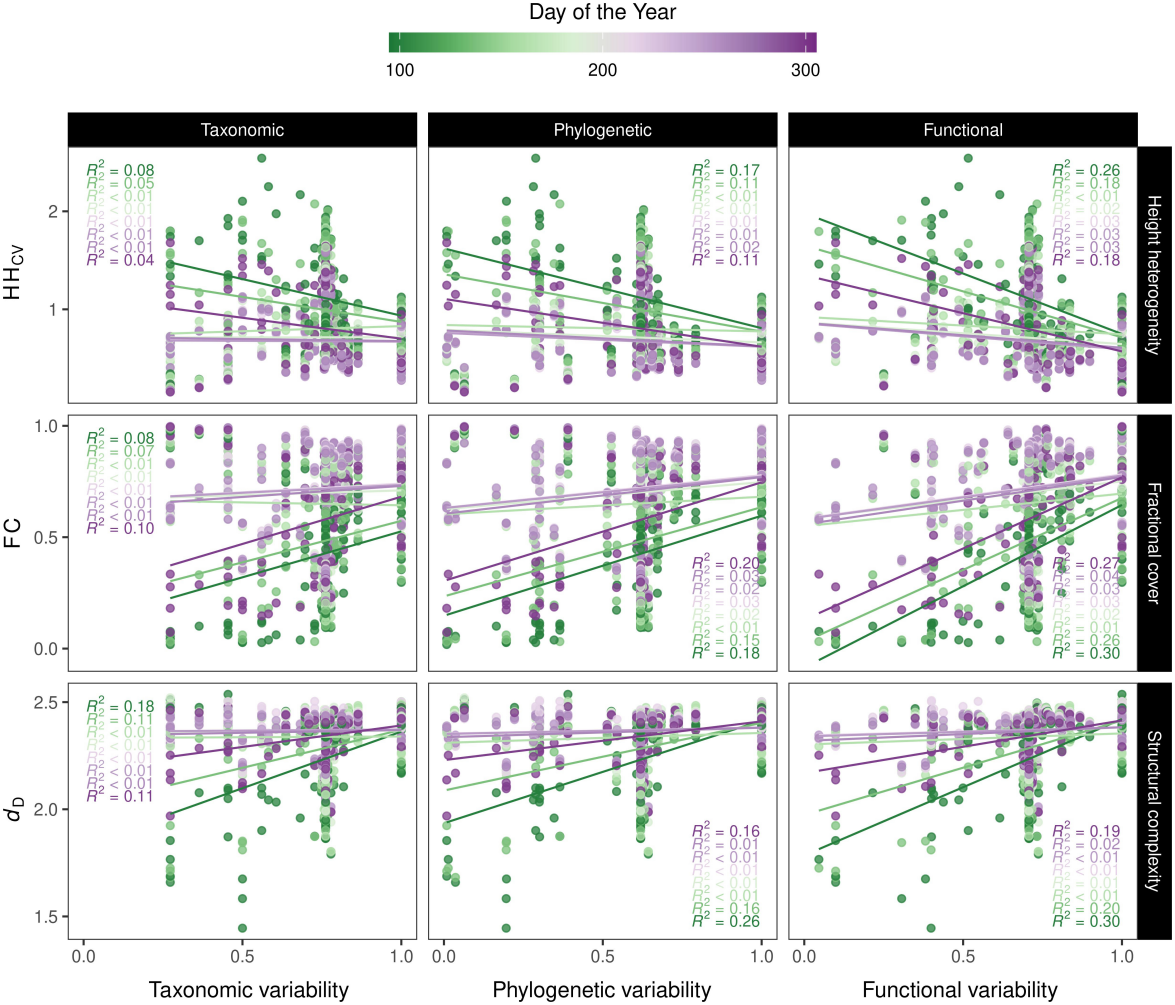
Relationships of taxonomic, phylogenetic, and functional variability with LiDAR‐derived metrics at different observation periods during the growing season. HH_CV_ describes the coefficient of variation of canopy height heterogeneity, FC the fractional plant cover, and *d*_D_ the fractal dimension. Taxonomic variability is similar to phylogenetic variability, but the tree topology is calculated from branch lengths of unity. See Appendix S1: Figure S6 for the significance of the regression models.

**FIGURE 7:**
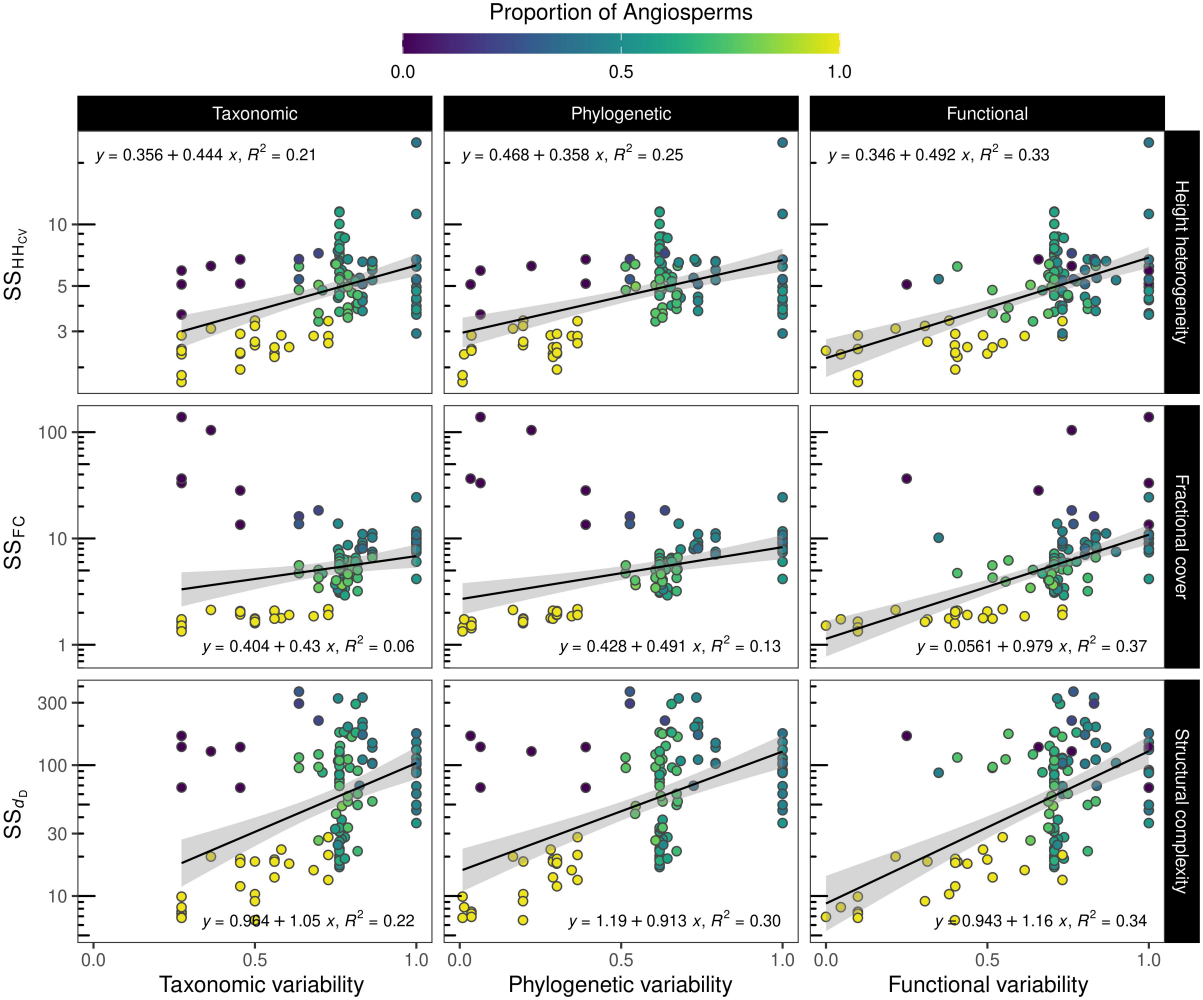
Association of different dimensions of variability with the structural stability (SS) of LiDAR‐derived metrics through the growing season. HH_CV_ describes the coefficient of variation of the canopy height heterogeneity, FC the fractional plant cover, and *d*_D_ the fractal dimension. Colors represent the proportion of angiosperm trees that were planted for each plot.

We found strong relationships between multiple dimensions of forest variability and structural metrics derived from LiDAR, revealing that communities composed of taxonomically, phylogenetically, and functionally dissimilar species had lower HH_CV_ but higher FC and *d*_D_ than plots composed of similar species (Figure 6; see Appendix S1: Figure S7 for greater detail). In contrast to diversity, where the relationships were better explained at the peak of the growing season (e.g., Figure 4), relationships between variability and forest structural metrics were better explained in observation periods close to the beginning and end of the growing season (i.e., leaves flushing and senescing). The variance explained by these relationships was also generally low (≤ 30%) and nonsignificant in most cases. These findings were supported by linear mixed models that revealed that observation periods and their interaction with dimensions of variability had a significant effect on the evaluated relationships (Appendix S1: Table S4). In addition, dimensions of variability were inversely associated with LiDAR‐derived metrics and explained a higher proportion of variance in most of the linear models in comparison with diversity metrics. When comparing dimensions of variability, our results indicated that functional variability within communities appeared to better explain changes in LiDAR‐derived metrics than phylogenetic or taxonomic variability (Figure 6; Appendix S1: Table S4). We further found that dimensions of variability consistently explained SS such that increases in variability were significantly associated with increases in SS_HHcv_, SS_FC_, and SS_*d*D_ (Figure 7). The coefficient of determination was higher for the relationships between dimensions of variability and SS than for the relationships between dimensions of diversity and SS (i.e., Figure 7 vs. Figure 5).

### Relationships with NBE

**FIGURE 8:**
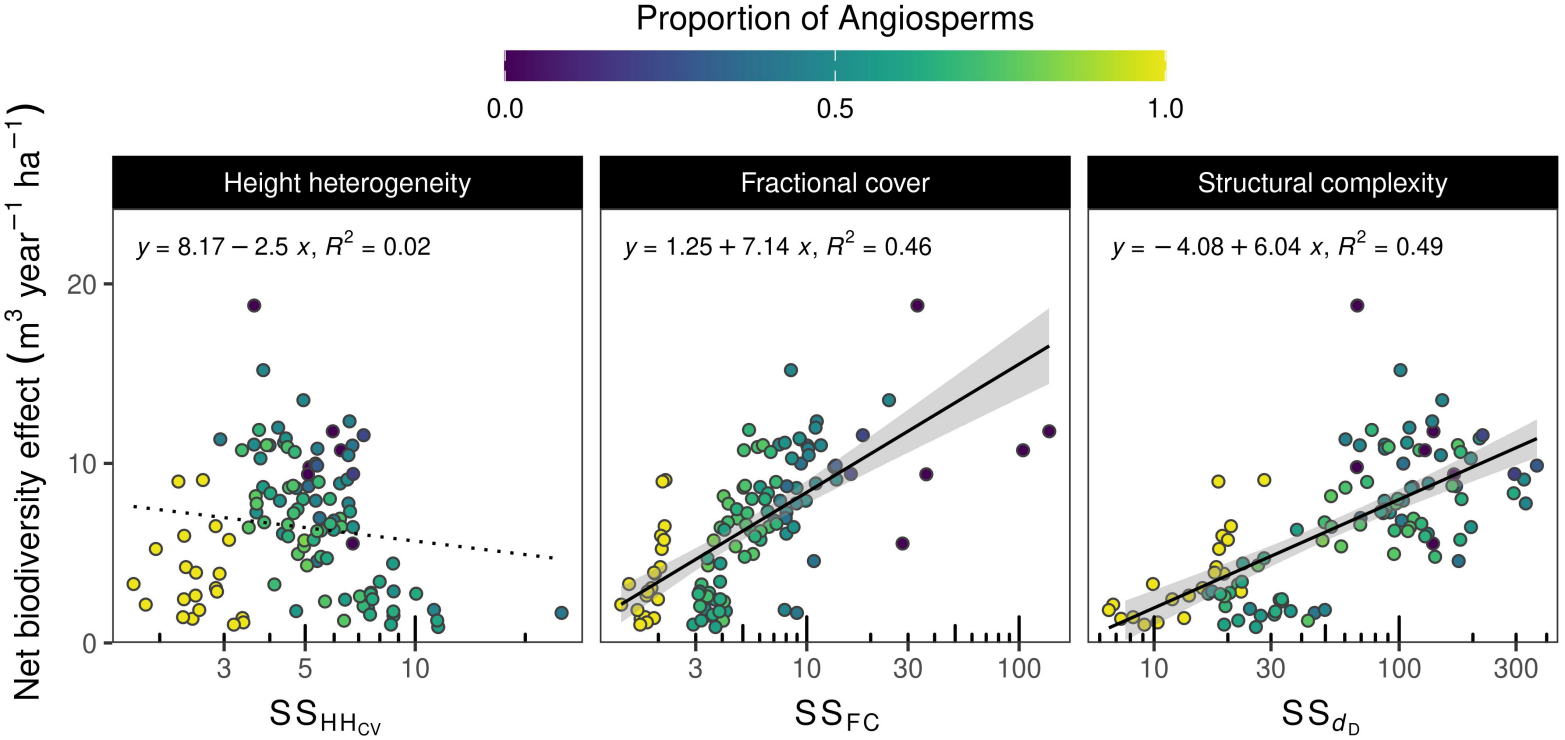
Relationships between seasonal structural stability (SS) of LiDAR‐derived metrics and the net biodiversity effect (NBE) of annual wood productivity. HH_CV_ describes the coefficient of variation of the canopy height heterogeneity, *FC* the fractional plant cover, and *d*_D_ the fractal dimension. Colors represent the proportion of angiosperm trees that were planted for each plot. See Appendix S1: Figure S8 for a description of the complementary and selection effects.

We found significant relationships between the SS of LiDAR‐derived metrics and NBEs. SS of forest complexity (SS_*d*D_) and fractional plant cover (SS_FC_)—but not SS_HHcv_—positively explained NBE (Figure 8). Increases in NBE associated with increasing structural stability (i.e., SS_*d*D_ and SS_FC_) were predominantly caused by complementarity effects rather than selection effects according to the coefficient of determination (Appendix S1: Figure S8). Moreover, NBE appeared to be negatively associated with species richness and FD (Appendix S1: Figure S9), but positively related to dimensions of variability (Figure S10). In addition, plots composed of a higher proportion of angiosperms presented lower NBE than those composed of a higher proportion of gymnosperms (Appendix S1: Figure S11). Although diversity, variability, and the proportion of angiosperm trees planted had an association with NBE, the variance explained by these relationships was not as high as the variance explained by the SS of forest complexity and fractional cover (i.e., SS_*d*D_ and SS_FC_) (i.e., Figure 8).

**FIGURE 9:**
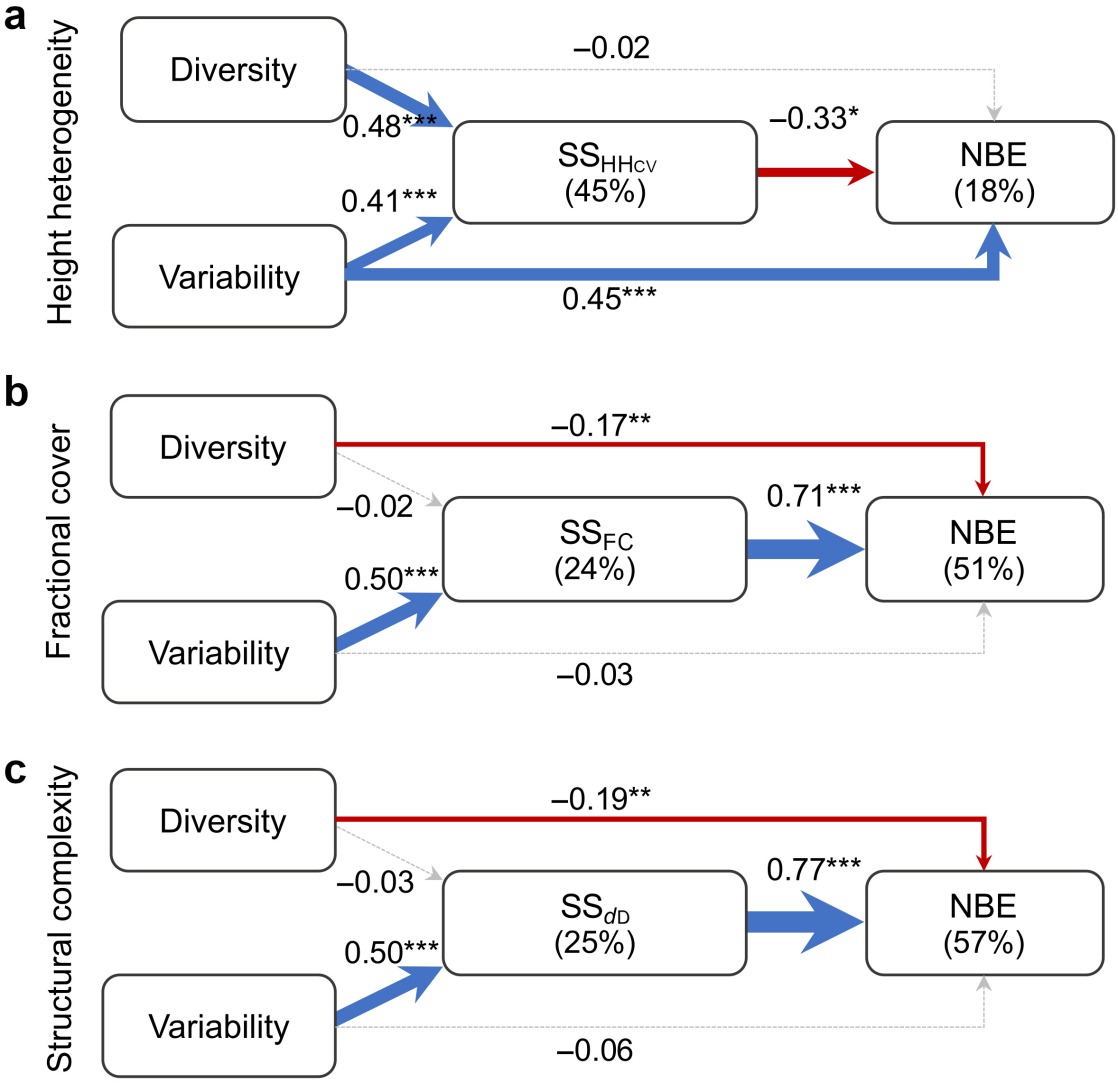
Structural equation models describing the paths of how multiple dimensions of diversity and variability are associated with the structural stability (SS) across the growing season, and in turn, explain the net biodiversity effect (NBE) of annual wood productivity (i.e., overyielding). Values next to the arrows represent the standardized coefficients and their significance, while values between parentheses are coefficient of determination. The thickness of the arrows is defined by the standardized coefficients, while their color indicates positive (blue), negative (red), or non‐effect (gray) association. The SS is described by the inverse of the coefficient of variation canopy height heterogeneity (SS_HHcv_), FC the fractional plant cover (SS_FC_), and *d*_D_ the fractal dimension (SS_*d*D_). Coefficients associated with the latent and regression variables can be found in Appendix S1: Tables S5 and S6.

Results of our SEM (Figure 9; Appendix S1: Tables S4 and S5) that incorporated latent variables to understand NBE revealed that dimensions of variability explained SS, which in turn explained NBE. Dimensions of diversity were only associated with SS in models based on SS_HHcv_ (Figure 9a) and appeared to be negatively related to NBE in models based on the stability of vegetation cover (SS_FC_) or of forest complexity (SS_*d*D_) (Figure 9b,c). Models based on SS_FC_ and SS_*d*D_ were closely linked to NBE, explaining up to 51% and 67% of the variance in NBE, respectively (Figure 9b,c). Changes in SS_*d*D_ and SS_FC_ were explained by variability, not diversity. Furthermore, the SEM based on high heterogeneity (SS_HHcv_) did not explain more than 20% of the variance in NBE, indicating that dimensions of variability were more important for explaining NBE than SS_HHcv_. However, in this model, both diversity and variability explained 45% of the variance in SS_HHcv_. Based on the standardized latent variables (Appendix S1: Table S4), FD was more important in explaining dimensions of diversity, while phylogenetic variability was more important in explaining dimensions of variability.

## DISCUSSION

In young stands of a large‐scale tree diversity experiment, we showed that multiple dimensions of forest diversity and variability explain remotely sensed metrics of forest structure and stability across the season. We also demonstrated that forest SS through the growing season was positively associated with NBEs on forest productivity. This study highlights emerging opportunities to understand the role of tree biodiversity in determining seasonal changes in forest structure and their consequences for ecosystem functions using remote sensing. Our work also shows that seasonal stability in forest structure is an emergent property of species interactions within communities that are coupled with ecosystem productivity.

### Relationships between forest volume and structural metrics derived from LiDAR

Our analyses revealed that LiDAR‐derived metrics of forest structure (i.e., HH_CV_, FC, and fractal dimension) were tightly coupled with changes in wood volume (e.g., Figure 2). These results indicate our remotely sensed metrics are reliable estimators of ground‐based observations of forest properties and are appropriate for detecting NBEs that emerge in this system. Despite this, our linear mixed models showed that the interaction of observation period with structural variables can affect the relationships between remotely sensed structure and both wood volume and NBE. Consequently, we find that measuring seasonal changes in forest community structure is critical to making inferences about forest function from remotely sensed observations. The interaction between observation period and forest structure may result from differences in plant strategies—which differ among deciduous broad‐leaved angiosperms and evergreen needle‐leaved gymnosperms—or in the timing of phenological events among species across the experiment. Differences in environmental conditions (e.g., wind) between the surveys may also cause changes in LiDAR estimations of forest structure. In addition, our results show that increases in the seasonal stability of structural complexity (i.e, SS_*d*D_) and FC (i.e., SS_FC_) were associated with increases in wood volume. These outcomes suggest that high seasonal SS may emerge as temperate forests mature.

### Association of multiple dimensions of diversity and composition with structural metrics derived from LiDAR and its seasonal stability

Our results support, to some extent, the premise that multiple dimensions of diversity explain remotely sensed metrics of forest structure. Overall, the observed association of height heterogeneity (HH_CV_) with species richness was consistent with the canopy heterogeneity hypothesis (Torresani et al., 2020) in which tree species richness is expected to enhance canopy heterogeneity. We expand on this hypothesis, suggesting that phylogenetic and FD are also likely to enhance canopy heterogeneity. Moreover, increases in diversity in this young forest experiment appeared to lead to communities that were more open and less structurally complex. This is an unexpected result because increases in diversity are expected to minimize space between trees through crown complementary while enhancing light interception (Williams et al., 2017, 2021). However, in our study, HH_CV_ and fractional cover declined, while forest complexity increased when variability increased. These contradictory trends between diversity and variability for phylogenetic and functional dimensions call into question the usefulness of the LiDAR structural metrics to predict forest biodiversity using a single observation period.

While diversity and variability showed contrasting relationships with remotely sensed structural metrics, we found that increases in both measures of biodiversity consistently showed positive associations with the stability of remotely sensed structural metrics across the season. We did not find direct evidence that observed wood volume was positively associated with diversity (Appendix S1: Figure S12), and neither measurement of biodiversity was related to species abundance (as determined by species volume). Consequently, our results appear to indicate that multiple dimensions of diversity and variability by themselves drive the stability of forest structure across the growing season. Forest diversity and variability can drive SS across the season due to the potential complementarity in structural strategies and changes of phenological rhythms among species across the season within forest mixtures. These factors may also explain why variability was more important in explaining differences in SS than diversity on forest complexity. In mixtures with low species richness, variability is likely to better disentangle differences among species than metrics of diversity because it highlights contrasting resource use strategies, particularly over time. In this regard, communities composed of phylogenetically or functionally different species resulted in higher SS than communities composed of similar species. Furthermore, our comparisons of multiple dimensions of diversity and variability indicated that the phylogenetic metrics (i.e., PD or phylogenetic variability) and, to some extent, functional metrics (i.e., FD or functional variability) were more important in describing changes in SS across the season than the taxonomic metrics (i.e., species richness or taxonomic variability) (e.g., Figure 9 or Appendix S1: Figure S12).

### Remotely sensed seasonal SS as an integrative metric to describe NBEs

Our results indicated that seasonal SS, explained by multiple dimensions of diversity and variability, was likely to enhance NBE on productivity in forest communities. Niche complementarity in resource use among species is often proposed as the driver explaining overyielding (Kelty, 1992; Tilman et al., 2001). Several mechanisms enhance NBE, including tree crown complementary, increased light interception and efficiency of light use, and temporal resource partitioning. Our remote estimates of seasonal SS integrate all these potential complementarity mechanisms that help to explain NBE. For instance, structural complexity (*d*_D_) indirectly describes how well tree crowns fit together in space, and thus enhance NBE, as assemblages that have higher and more stable complexity (Guzmán et al., 2020) are those with structures that homogeneously fill in their available three‐dimensional space over time. Likewise, high fractional cover (FC) and its stability are likely to capture enhanced light interception, given that FC is associated with the probability of laser pulses being intercepted by trees before reaching the forest floor. Despite being a young forest experiment, we further expect temporal resource partitioning to be captured by LiDAR‐based measures of seasonal SS, since it integrates information about the phenological variation changes among species that are linked to intrinsic growth strategies (i.e., fast‐ or slow‐growing). The importance of temporal resource partitioning for biodiversity effects on productivity in temperate mixed forest is supported by Lu et al. (2016) who suggested that differences in leaf phenology and shade tolerance lead to increases in overyielding in evergreen‐deciduous mixtures but not in deciduous–deciduous mixtures. Evergreen–deciduous mixtures (i.e., gymnosperms–angiosperms in our case) are expected to capture and use resources differently across the season, helping to explain why complementarity, and not selection effects, is more tightly coupled with SS (e.g., Appendix S1: Figure S8). Distantly related groups of species, in general, tend to capture and use resources in contrasting ways, reducing competition and contributing to their complementarity. In long‐term grassland experiments, for example, communities composed of distantly related species buffered ecosystems against environmental fluctuation variations, resulting in greater stability of productivity (Cadotte et al., 2012). Moreover, HH_CV_ only represents the changes at the top of the canopy but does not describe the three‐dimensionality of forest structure, which may explain why this metric was not as clearly linked to NBE on productivity.

### Remote sensing of forest SS as a tool to evaluate ecological processes

Remotely sensed forest SS through the season appears to integrate several drivers of overyielding. Therefore, seasonal SS may help to discern the role of diversity in other ecological processes within forest communities, such as alteration of microclimates, forest resilience, and ecosystem functions that generate important services. For instance, given the strong influence of forest structure on microclimate, it is likely that SS promoted by diversity and variability alters the temporal fluctuations in forest microclimate, buffering communities from environmental extremes (Potter et al., 2001). The temporal fluctuations in microclimate resulting from SS are also likely to trigger changes in belowground diversity (Lang et al., 2023) and, therefore, the availability of soil nutrients. In addition, structurally stable and complex communities may create optimal conditions that enable other plant species to colonize available spaces (Walter et al., 2021) and enhance belowground diversity (Lang et al., 2023). Currently, there are a large number of metrics that can be derived from LiDAR (e.g., Atkins, Bhatt, et al., 2023), many of which are affected by spatial scales (Atkins et al. 2023b) or are redundant in terms of their ecological meaning. Therefore, the future integration of LiDAR with ecology should focus on metrics that are not dependent on spatial or temporal scales and help to evaluate multiple dimensions of biodiversity.

## CONCLUSIONS

Here we provide empirical evidence that taxonomic, phylogenetic, and functional dimensions of diversity and variability (i.e., the degree which species in a community are related) have an important association with forest structural metrics derived from UAV‐LiDAR in a young tree diversity experiment. Most importantly, we show that increases in diversity and variability have a positive relationship with the temporal stability of forest structure throughout the growing season. We further demonstrate that increases in the SS of forest complexity (i.e., fractal geometry) and FC explain increases in NBEs on productivity. These findings highlight remotely sensed SS as an integrative metric that encompasses several drivers of NBEs on forest productivity. Our study demonstrates the potential of coupling remote sensing and ecology to explore the role of forest SS in shaping ecological processes.

## AUTHOR CONTRIBUTIONS

J. Antonio Guzmán Q. and Jeannine Cavender‐Bares designed the research. J. Antonio Guzmán Q. conducted the UAV‐LiDAR surveys with the assistance of Jeannine Cavender‐Bares. J. Antonio Guzmán Q. processed, analyzed, and visualized the data. Maria H. Park drafted the conceptual figure with inputs from J. Antonio Guzmán Q. and Jeannine Cavender‐Bares. J. Antonio Guzmán Q. drafted the manuscript with inputs from Maria H. Park, Laura J. Williams, and Jeannine Cavender‐Bares. Funding sources come through Jeannine Cavender‐Bares. All authors contributed intellectually to the project and to the editing directions of the manuscript.

## CONFLICT OF INTEREST STATEMENT

The authors declare no conflicts of interest.

## Supporting information


Appendix S1.

## Data Availability

FAB2 forest inventory data (Cavender‐Bares, 2025) are available on the Environmental Data Initiative (EDI) Data Portal: https://doi.org/10.6073/pasta/d13bc1c1f168662be4ae2803a776935e. The processed point clouds from the UAV‐LiDAR surveys (Guzmán et al., 2025a) are available on Dryad: https://doi.org/10.5061/dryad.jdfn2z3hk. All the code (Guzmán et al., 2025b) used to process, analyze, and visualize data is available on Zenodo: https://doi.org/10.5281/zenodo.14645681.

## References

[ecy70055-bib-0001] Aphalo, P. J. 2023. “ggpmisc: Miscellaneous Extensions to ‘ggplot2’.” R package version 0.6.1. https://CRAN.R-project.org/package=ggpmisc

[ecy70055-bib-0002] Atkins, J. W. , P. Bhatt , L. Carrasco , E. Francis , J. E. Garabedian , C. R. Hakkenberg , B. S. Hardiman , et al. 2023. “Integrating Forest Structural Diversity Measurement into Ecological Research.” Ecosphere 14: e4633.

[ecy70055-bib-0003] Atkins, J. W. , J. Costanza , K. M. Dahlin , M. P. Dannenberg , A. J. Elmore , M. C. Fitzpatrick , C. R. Hakkenberg , et al. 2023. “Scale Dependency of Lidar‐Derived Forest Structural Diversity.” Methods in Ecology and Evolution 14: 708–723.

[ecy70055-bib-0004] Bates, D. , M. Mächler , B. Bolker , and S. Walker . 2015. “Fitting Linear Mixed‐Effects Models Using lme4.” Journal of Statistical Software 67: 1–48.

[ecy70055-bib-0005] Boucher, P. B. , E. G. Hockridge , J. Singh , and A. B. Davies . 2023. “Flying High: Sampling Savanna Vegetation with UAV‐Lidar.” Methods in Ecology and Evolution 14: 1668–1686.

[ecy70055-bib-0006] Cadotte, M. W. , R. Dinnage , and D. Tilman . 2012. “Phylogenetic Diversity Promotes Ecosystem Stability.” Ecology 93: S223–S233.

[ecy70055-bib-0007] Cavender‐Bares, J. 2025. “FAB2_sapling_volume_2021–2022 in Forest and Biodiversity 2: A Tree Diversity Experiment to Understand the Consequences of Multiple Dimensions of Diversity and Composition for Long‐Term Ecosystem Function and Resilience ver 1.” Environmental Data Initiative. 10.6073/pasta/d13bc1c1f168662be4ae2803a776935e.

[ecy70055-bib-0008] Cavender‐Bares, J. , J. J. Grossman , Q. J. A. Guzmán , S. E. Hobbie , M. A. Kaproth , S. Kothari , C. N. Lapadat , R. A. Montgomery , and M. Park . 2024. “Forest and Biodiversity 2: A Tree Diversity Experiment to Understand the Consequences of Multiple Dimensions of Diversity and Composition for Long‐Term Ecosystem Function and Resilience.” Methods in Ecology and Evolution 15: 2400–2414.

[ecy70055-bib-0009] Cavender‐Bares, J. , F. D. Schneider , M. J. Santos , A. Armstrong , A. Carnaval , K. M. Dahlin , L. Fatoyinbo , et al. 2022. “Integrating Rremote Sensing with Ecology and Evolution to Advance Biodiversity Conservation.” Nature Ecology & Evolution 6: 506–519. 35332280 10.1038/s41559-022-01702-5

[ecy70055-bib-0010] Chao, A. , C.‐H. Chiu , and L. Jost . 2014. “Unifying Species Diversity, Phylogenetic Diversity, Functional Diversity, and Related Similarity and Differentiation Measures through Hill Numbers.” Annual Review of Ecology, Evolution, and Systematics 45: 297–324.

[ecy70055-bib-0011] Clarke, K. R. , and R. M. Warwick . 1998. “A Taxonomic Distinctness Index and its Statistical Properties.” Journal of Applied Ecology 35: 523–531.

[ecy70055-bib-0012] del Río, M. , H. Pretzsch , R. Ruiz‐Peinado , H. Jactel , L. Coll , M. Löf , J. Aldea , et al. 2022. “Emerging Stability of Forest Productivity by Mixing Two Species Buffers Temperature Destabilizing Effect.” Journal of Applied Ecology 59: 2730–2741.

[ecy70055-bib-0013] Dolezal, J. , P. Fibich , J. Altman , K. Takahashi , and T. Hara . 2024. “Diversity Effects and Compensatory Dynamics Drive Productivity and Stability in Temperate Old‐Growth Forests.” Journal of Ecology 112: 2249–2263.

[ecy70055-bib-0014] Ehbrecht, M. , P. Schall , C. Ammer , and D. Seidel . 2017. “Quantifying Stand Structural Complexity and its Relationship with Forest Management, Tree Species Diversity and Microclimate.” Agricultural and Forest Meteorology 242: 1–9.

[ecy70055-bib-0015] Guzmán, J. , R. Hernandez , and A. Sanchez‐Azofeifa . 2021. “rTLS: Tools to Process Point Clouds Derived from Terrestrial Laser Scanning.” R package version 0.2.5. https://CRAN.R-project.org/package=rTLS

[ecy70055-bib-0016] Guzmán, J. A. , M. H. Park , L. J. Williams , and J. Cavender‐Bares . 2025a. “UAV‐LiDAR Point Clouds from the Forest and Biodiversity Experiment 2 (2022).” Dataset. Dryad. 10.5061/dryad.jdfn2z3hk

[ecy70055-bib-0017] Guzmán, J. A. , M. H. Park , L. J. Williams , and J. Cavender‐Bares . 2025b. “Code for processing UAV‐LiDAR on Forest and Biodiversity Experiments. In Seasonal structural stability promoted by forest diversity and composition explains overyielding (v0.1.0).” Zenodo. 10.5281/zenodo.14645681 PMC11911966 40091772

[ecy70055-bib-0018] Guzmán, J. A. , I. Sharp , F. Alencastro , and G. A. Sánchez‐Azofeifa . 2020. “On the Relationship of Fractal Geometry and Tree–Stand Metrics on Point Clouds Derived from Terrestrial Laser Scanning.” Methods in Ecology and Evolution 11: 1309–1318.

[ecy70055-bib-0019] Helmus, M. R. , T. J. Bland , C. K. Williams , and A. R. Ives . 2007. “Phylogenetic Measures of Biodiversity.” The American Naturalist 169: E68–E83. 10.1086/511334 17230400

[ecy70055-bib-0020] Hooper, D. U. , F. S. Chapin , J. J. Ewel , A. Hector , P. Inchausti , S. Lavorel , J. H. Lawton , et al. 2005. “Effects of Biodiversity on Ecosystem Functioning: A Consensus of Current Knowledge.” Ecological Monographs 75: 3–35.

[ecy70055-bib-0021] Isenburg, M. 2014. “LAStools: Efficient LiDAR Processing Software. rapidlasso.” Version 2.0.2. https://github.com/LAStools/LAStools

[ecy70055-bib-0022] Jin, Y. , and H. Qian . 2022. “V.PhyloMaker2: An Updated and Enlarged R Package that Can Generate Very Large Phylogenies for Vascular Plants.” Plant Diversity 44: 335–339. 35967255 10.1016/j.pld.2022.05.005 PMC9363651

[ecy70055-bib-0023] Jost, L. 2006. “Entropy and Diversity.” Oikos 113: 363–375.

[ecy70055-bib-0024] Juchheim, J. , M. Ehbrecht , P. Schall , C. Ammer , and D. Seidel . 2019. “Effect of Tree Species Mixing on Stand Structural Complexity.” Forestry: An International Journal of Forest Research 93: 75–83.

[ecy70055-bib-0025] Kelty, M. J. 1992. “Comparative Productivity of Monocultures and Mixed‐Species Stands.” In The Ecology and Silviculture of Mixed‐Species Forests, edited by M. J. Kelty , B. C. Larson , and C. D. Oliver , 125–141. Dordrecht: Springer Netherlands.

[ecy70055-bib-0026] Kembel, S. W. , P. D. Cowan , M. R. Helmus , W. K. Cornwell , H. Morlon , D. D. Ackerly , S. P. Blomberg , and C. O. Webb . 2010. “Picante: R Tools for Integrating Phylogenies and Ecology.” Bioinformatics 26: 1463–1464. 20395285 10.1093/bioinformatics/btq166

[ecy70055-bib-0027] Kunert, N. , L. Schwendenmann , C. Potvin , and D. Hölscher . 2012. “Tree Diversity Enhances Tree Transpiration in a Panamanian Forest Plantation.” Journal of Applied Ecology 49: 135–144.

[ecy70055-bib-0028] Kunz, M. , A. Fichtner , W. Härdtle , P. Raumonen , H. Bruelheide , and G. Von Oheimb . 2019. “Neighbour Species Richness and Local Structural Variability Modulate Aboveground Allocation Patterns and Crown Morphology of Individual Trees.” Ecology Letters 22: 2130–2140. 31625279 10.1111/ele.13400

[ecy70055-bib-0029] Lang, A. K. , E. A. LaRue , S. N. Kivlin , J. D. Edwards , R. P. Phillips , J. Gallion , N. Kong , et al. 2023. “Forest Structural Diversity is Linked to Soil Microbial Diversity.” Ecosphere 14: e4702.

[ecy70055-bib-0030] Li, D. 2018. “hillR: Taxonomic, Functional, and Phylogenetic Diversity and Similarity through Hill Numbers.” Journal of Open Source Software 3: 1041.

[ecy70055-bib-0058] Liu, X. , Q. Ma , X. Wu , T. Hu , G. Dai , J. Wu , S. Tao , et al. 2022. “Nonscalability of Fractal Dimension to Quantify Canopy Structural Complexity from Individual Trees to Forest Stands.” Journal of Remote Sensing 2022: 0001.

[ecy70055-bib-0057] Loreau, M. , and H. Hector . 2021. “Partitioning Selection and Complementarity in Biodiversity Experiments.” Nature 412: 72–76. 10.1038/35083573 11452308

[ecy70055-bib-0031] Loreau, M. , M. Barbier , E. Filotas , D. Gravel , F. Isbell , S. J. Miller , J. M. Montoya , et al. 2021. “Biodiversity as Insurance: From Concept to Measurement and Application.” Biological Reviews 96: 2333–2354. 34080283 10.1111/brv.12756 PMC8519139

[ecy70055-bib-0032] Lu, H. , G. M. J. Mohren , J. Den Ouden , V. Goudiaby , and F. J. Sterck . 2016. “Overyielding of Temperate Mixed Forests Occurs in Evergreen–Deciduous but Not in Deciduous–Deciduous Species Mixtures over Time in The Netherlands.” Forest Ecology and Management 376: 321–332.

[ecy70055-bib-0033] Messier, C. , J. Bauhus , R. Sousa‐Silva , H. Auge , L. Baeten , N. Barsoum , H. Bruelheide , et al. 2022. “For the Sake of Resilience and Multifunctionality, Let's Diversify Planted Forests!” Conservation Letters 15: e12829.

[ecy70055-bib-0034] Oksanen, J. , G. L. Simpson , F. G. Blanchet , R. Kindt , P. Legendre , P. R. Minchin , R. B. O'Hara , et al. 2022. “vegan: Community Ecology Package.”

[ecy70055-bib-0035] Paradis, E. , J. Claude , and K. Strimmer . 2004. “APE: Analyses of Phylogenetics and Evolution in R Language.” Bioinformatics 20: 289–290. 14734327 10.1093/bioinformatics/btg412

[ecy70055-bib-0036] Potter, B. E. , R. M. Teclaw , and J. C. Zasada . 2001. “The Impact of Forest Structure on Near‐Ground Temperatures during Two Years of Contrasting Temperature Extremes.” Agricultural and Forest Meteorology 106: 331–336.

[ecy70055-bib-0037] R Core Team . 2023. R: A Language and Environment for Statistical Computing. Vienna, Austria: R Foundation for Statistical Computing.

[ecy70055-bib-0038] Ray, T. , B. M. Delory , R. Beugnon , H. Bruelheide , S. Cesarz , N. Eisenhauer , O. Ferlian , J. Quosh , G. Von Oheimb , and A. Fichtner . 2023. “Tree Diversity Increases Productivity through Enhancing Structural Complexity across Mycorrhizal Types.” Science Advances 9: eadi2362. 37801499 10.1126/sciadv.adi2362 PMC10558120

[ecy70055-bib-0039] Robinson, C. , S. Saatchi , D. Clark , J. Hurtado Astaiza , A. Hubel , and T. Gillespie . 2018. “Topography and Three‐Dimensional Structure Can Estimate Tree Diversity along a Tropical Elevational Gradient in Costa Rica.” Remote Sensing 10: 629.

[ecy70055-bib-0040] Rosseel, Y. 2012. “lavaan: An *R* Package for Structural Equation Modeling.” Journal of Statistical Software 48: 1–36.

[ecy70055-bib-0041] Roussel, J.‐R. , D. Auty , N. C. Coops , P. Tompalski , T. R. H. Goodbody , A. S. Meador , J.‐F. Bourdon , F. De Boissieu , and A. Achim . 2020. “lidR: An R Package for Analysis of Airborne Laser Scanning (ALS) Data.” Remote Sensing of Environment 251: 112061.

[ecy70055-bib-0042] Schnabel, F. , X. Liu , M. Kunz , K. E. Barry , F. J. Bongers , H. Bruelheide , A. Fichtner , et al. 2021. “Species Richness Stabilizes Productivity Via Asynchrony and Drought‐Tolerance Diversity in a Large‐Scale Tree Biodiversity Experiment.” Science Advances 7: eabk1643. 34919425 10.1126/sciadv.abk1643 PMC8682986

[ecy70055-bib-0043] Schnabel, F. , J. A. Schwarz , A. Dănescu , A. Fichtner , C. A. Nock , J. Bauhus , and C. Potvin . 2019. “Drivers of Productivity and its Temporal Stability in a Tropical Tree Diversity Experiment.” Global Change Biology 25: 4257–4272. 31486578 10.1111/gcb.14792

[ecy70055-bib-0044] Seidel, D. , C. Leuschner , C. Scherber , F. Beyer , T. Wommelsdorf , M. J. Cashman , and L. Fehrmann . 2013. “The Relationship between Tree Species Richness, Canopy Space Exploration and Productivity in a Temperate Broad‐Leaf Mixed Forest.” Forest Ecology and Management 310: 366–374.

[ecy70055-bib-0045] Smith, M. N. , S. C. Stark , T. C. Taylor , M. L. Ferreira , E. De Oliveira , N. Restrepo‐Coupe , S. Chen , et al. 2019. “Seasonal and Drought‐Related Changes in Leaf Area Profiles Depend on Height and Light Environment in an Amazon Forest.” New Phytologist 222: 1284–1297. 30720871 10.1111/nph.15726

[ecy70055-bib-0046] Smith, S. A. , and J. W. Brown . 2018. “Constructing a Broadly Inclusive Seed Plant Phylogeny.” American Journal of Botany 105: 302–314. 29746720 10.1002/ajb2.1019

[ecy70055-bib-0047] Tilman, D. , P. B. Reich , J. Knops , D. Wedin , T. Mielke , and C. Lehman . 2001. “Diversity and Productivity in a Long‐Term Grassland Experiment.” Science 294: 843–845. 11679667 10.1126/science.1060391

[ecy70055-bib-0048] Torresani, M. , D. Rocchini , A. Alberti , V. Moudrý , M. Heym , E. Thouverai , P. Kacic , and E. Tomelleri . 2023. “LiDAR GEDI Derived Tree Canopy Height Heterogeneity Reveals Patterns of Biodiversity in Forest Ecosystems.” Ecological Informatics 76: 102082. 37662896 10.1016/j.ecoinf.2023.102082 PMC10316066

[ecy70055-bib-0049] Torresani, M. , D. Rocchini , R. Sonnenschein , M. Zebisch , H. C. Hauffe , M. Heym , H. Pretzsch , and G. Tonon . 2020. “Height Variation Hypothesis: A New Approach for Estimating Forest Species Diversity with CHM LiDAR Data.” Ecological Indicators 117: 106520.

[ecy70055-bib-0050] Turner, W. 2014. “Sensing Biodiversity.” Science 346: 301–302. 25324372 10.1126/science.1256014

[ecy70055-bib-0051] Walter, J. A. , A. E. L. Stovall , and J. W. Atkins . 2021. “Vegetation Structural Complexity and Biodiversity in the Great Smoky Mountains.” Ecosphere 12: e03390.

[ecy70055-bib-0052] Warton, D. I. , R. A. Duursma , D. S. Falster , and S. Taskinen . 2012. “Smatr 3– An R Package for Estimation and Inference about Allometric Lines.” Methods in Ecology and Evolution 3: 257–259.

[ecy70055-bib-0053] Wickham, H. 2016. ggplot2: Elegant Graphics for Data Analysis. New York, NY: Springer‐Verlag.

[ecy70055-bib-0054] Williams, L. J. , E. E. Butler , J. Cavender‐Bares , A. Stefanski , K. E. Rice , C. Messier , A. Paquette , and P. B. Reich . 2021. “Enhanced Light Interception and Light Use Efficiency Explain Overyielding in Young Tree Communities.” Ecology Letters 24: 996–1006. 33657676 10.1111/ele.13717

[ecy70055-bib-0055] Williams, L. J. , A. Paquette , J. Cavender‐Bares , C. Messier , and P. B. Reich . 2017. “Spatial Complementarity in Tree Crowns Explains Overyielding in Species Mixtures.” Nature Ecology & Evolution 1: 63. 28812675 10.1038/s41559-016-0063

[ecy70055-bib-0056] Yachi, S. , and M. Loreau . 1999. “Biodiversity and Ecosystem Productivity in a Fluctuating Environment: The Insurance Hypothesis.” Proceedings of the National Academy of Sciences 96: 1463–1468. 10.1073/pnas.96.4.1463 PMC15485 9990046

