# Supplementary material for: Seasonal structural stability promoted by forest diversity and composition explains overyielding
Source: Ecology. 2025 Mar 17;106(3):e70055. doi: 10.1002/ecy.70055 (PMC11911966; doi:10.1002/ecy.70055)
Supplement: Supplementary file 1 — Appendix S1. [file ECY-106-e70055-s001.pdf]

Ecology

Appendix S1

Seasonal structural stability promoted by forest diversity and composition explains overyielding

J. Antonio Guzmán Q., Maria H. Park, Laura J. Williams, Jeannine Cavender-Bares

Appendix S1: LiDAR ground classification and height normalization

We applied a series of steps to ensure an optimal classification of points as ground/non-ground and their posterior normalization by height. These steps were inspired by (but not identical to) Mattie (2022), and are an attempt to correct for the 'thickness' of point clouds collected by the Zenmuse L1 LiDAR using LAStools Isenburg (2014). For this we first selected candidates for ground points using percentiles between 10% and 20% according to point height by applying consecutive different grid sizes (20, 40, 60 cm). At each grid size these candidate low points were classified as model points (Class 8) using *lasthin* function. On the resulting candidates, we then ran a ground classification through *lasground* function in which low ground points (Class 2) were defined. This low ground classification procedure was defined using 'coarse' and 'nature' settings avoiding spikes above 0.25 m for better performance. Using these low ground points we then ran a height normalization through *lasheight* function to replace point elevation by point height from the ground; providing height-normalized point clouds for further analysis.

Table S1. Characteristics and plant functional traits of species at the Forest and Biodiversity II Experiment.

Order	Family	Species	Wood density[1]	LMA[2]	Shade tolerance index[3]	Maximum height[4] (m)	RGR[5]
Sapindales	Sapindaceae	*Acer negundo*	0.42	37.04	3.47	18.59	0.039
Sapindales	Sapindaceae	*Acer rubrum*	0.49	71.09	3.44	19.81	0.379
Fagales	Betulaceae	*Betula papyrifera*	0.48	77.88	1.54	11.58	1.272
Pinales	Cupressaceae	*Juniperus virginiana*	0.44	333.33	1.28	14.63	0.975
Pinales	Pinaceae	*Pinus banksiana*	0.4	243.9	1.36	22.25	2.288
Pinales	Pinaceae	*Pinus resinosa*	0.41	294.12	1.89	25.91	1.619
Pinales	Pinaceae	*Pinus strobus*	0.34	121.92	3.21	27.13	1.722
Fagales	Fagaceae	*Quercus alba*	0.6	81.21	2.85	24.38	0.735
Fagales	Fagaceae	*Quercus ellipsoidalis*	0.59	90	2.49	23.16	0.722
Fagales	Fagaceae	*Quercus macrocarpa*	0.58	92.74	2.71	21.64	0.547
Fagales	Fagaceae	*Quercus rubra*	0.56	84.2	2.75	24.99	0.505
Malvales	Malvaceae	*Tilia americana*	0.32	60.81	3.98	23.77	0.724

[1] Wood density (g cm^{-3}) from Zanne et al. (2009).
[2] Leaf mass per area (m^2 kg^{-1}) from Grossman et al. (2017).
[3] Shade tolerance ranking from 1 (shade intolerant) to 5 (shade tolerant) from Niinemets and Valladares (2006).
[4] Maximum height (quantile 99%) of trees in inventories between 2018 to 2022 from the Forest Inventory and Analysis National Program across Minnesota, US.
[5] Mean relative growth rate (RGR, $year^{-1}$) of species on monocultures between 2013 to 2016 from the first Forest and Biodiversity Experiment at Cedar Creek Ecosystem Science Reserve, Minnesota, USA (Kothari et al. 2021).

Table S2. Linear mixed model statistics for comparing the wood volume and day of the year (DOY), and their interaction on LiDAR derived metrics. For the fixed effects, the upper values in each row represent the *F-ratio* of Analysis of Variance using Type II Wald F-tests, while lower values represent its significance. σ^2 describes the variance of the model, τ_{00} the variance of the random effects, ICC the interclass correlation coefficients, and *df* the degrees of freedom.

Predictors	LiDAR-derived metric		
	Height heterogeneity (HH_{CV})	Fractional cover (FC)	Structural complexity (d_D)
**Fixed effects**			
Volume (df = 1, 168)	771.47	1395.23	787.76
	$p < 0.001$	$p < 0.001$	$p < 0.001$
DOY (df = 1, 1188)	477.50	457.53	395.10
	$p < 0.001$	$p < 0.001$	$p < 0.001$
Interaction (df = 1, 1188)	33.59	3.30	303.81
	$p < 0.001$	$p < 0.001$	$p < 0.001$
**Random effects**			
σ^2	0.03	0.02	0.01
τ_{00} plot:block	0.02	0.00	0.00
ICC	0.38	0.20	0.29
Marginal R^2	0.70	0.73	0.68
Conditional R^2	0.81	0.78	0.77

Table S3. Linear mixed model statistics for evaluating the association of multiple dimensions of diversity, the day of the year (DOY), and their interaction with LiDAR derived metrics. For the fixed effects, the upper values in each row represent the *F-ratio* of Analysis of Variance using Type II Wald F-tests, while lower values represent its significance. ICC describes the intraclass correlation coefficients and *df* the degrees of freedom.

Dimensions of diversity	Effects	Predictors	LiDAR-derived metric		
			Height heterogeneity (HH_{CV})	Fractional cover (*FC*)	Structural complexity (d_D)
Taxonomic	Fixed	Species richness (*df* = 1, 168)	41.15<br>$p < 0.001$	20.69<br>$p < 0.001$	7.03<br>$p < 0.01$
		DOY (*df* = 1, 1188)	467.29<br>$p < 0.001$	458.00<br>$p < 0.001$	315.46<br>$p < 0.001$
		Interaction (*df* = 1, 1188)	7.47<br>$p < 0.01$	4.52<br>$p = 0.03$	1.56<br>$p = 0.08$
	Random	ICC	0.76	0.75	0.69
		Marginal R^2	0.22	0.15	0.09
		Conditional R^2	0.81	0.79	0.72
Phylogenetic	Fixed	*PD* (*df* = 1, 168)	18.72<br>$p < 0.001$	6.39<br>$p = 0.01$	0.62<br>$p = 0.43$
		DOY (*df* = 1, 1188)	464.62<br>$p < 0.001$	458.95<br>$p < 0.001$	322.11<br>$p < 0.001$
		Interaction (*df* = 1, 1188)	1.14<br>$p = 0.42$	6.99<br>$p < 0.001$	28.20<br>$p < 0.001$
	Random	ICC	0.78	0.76	0.70
		Marginal R^2	0.14	0.10	0.07
		Conditional R^2	0.81	0.78	0.72

Dimensions of diversity	Effects	Predictors	LiDAR-derived metric		
			Height heterogeneity (HH_{CV})	Fractional cover (*FC*)	Structural complexity (d_D)
Functional	Fixed	*FD* ($df = 1, 168$)	32.45	14.74	3.86
			$p < 0.001$	$p < 0.001$	$p = 0.05$
		DOY ($df = 1, 1188$)	466.23	458.55	317.15
			$p < 0.001$	$p < 0.001$	$p < 0.001$
		Interaction ($df = 1, 1188$)	4.81	5.96	9.49
			$p = 0.02$	$p = 0.02$	$p < 0.01$
	Random	ICC	0.77	0.75	0.69
		Marginal R^2	0.19	0.13	0.08
		Conditional R^2	0.81	0.79	0.72

Table S4. Linear mixed model statistics for evaluating the association of multiple dimensions of species variability, the day of the year (DOY), and their interaction with LiDAR derived metrics. For fixed effects, the upper values in each row represent the *F-ratio* of Analysis of Variance using Type II Wald F-tests, while lower values represent its significance. ICC describes the intraclass correlation coefficients and *df* the degrees of freedom.

Dimensions of variability	Effects	Predictors	LiDAR-derived metric		
			Height heterogeneity (HH_{CV})	Fractional cover (*FC*)	Structural complexity (d_D)
Taxonomic	Fixed	Species richness (*df* = 1, 115)	2.76<br>$p = 0.09$	5.14<br>$p = 0.02$	10.67<br>$p = 0.002$
		DOY (*df* = 1, 817)	404.64<br>$p < 0.001$	268.74<br>$p < 0.001$	197.99<br>$p < 0.001$
		Interaction (*df* = 1, 817)	16.86<br>$p < 0.001$	1.78<br>$p = 0.18$	77.23<br>$p < 0.001$
	Random	ICC	0.79	0.77	0.69
		Marginal R^2	0.10	0.09	0.14
		Conditional R^2	0.81	0.79	0.73
Phylogenetic	Fixed	*PD* (*df* = 1, 115)	8.44<br>$p < 0.01$	12.69<br>$p < 0.001$	16.84<br>$p < 0.001$
		DOY (*df* = 1, 817)	410.52<br>$p < 0.001$	269.67<br>$p < 0.001$	204.34<br>$p < 0.001$
		Interaction (*df* = 1, 817)	28.99<br>$p < 0.001$	3.37<br>$p = 0.07$	105.88<br>$p < 0.001$
	Random	ICC	0.79	0.76	0.69
		Marginal R^2	0.14	0.13	0.17
		Conditional R^2	0.82	0.79	0.74

Dimensions of variability	Effects	Predictors	LiDAR-derived metric		
			Height heterogeneity (HH_{CV})	Fractional cover (*FC*)	Structural complexity (d_D)
Functional	Fixed	*FD* ($df = 1, 115$)	14.21	18.03	21.89
			$p < 0.001$	$p < 0.001$	$p < 0.001$
		DOY ($df = 1, 817$)	416.23	272.22	208.93
			$p < 0.001$	$p < 0.001$	$p < 0.001$
		Interaction ($df = 1, 817$)	40.74	13.36	126.60
			$p < 0.001$	$p < 0.001$	$p < 0.001$
	Random	ICC	0.78	0.75	0.69
		Marginal R^2	0.17	0.17	0.20
		Conditional R^2	0.82	0.79	0.75

Table S5. Results from the structural regression models associated with the latent variables on models based on the forest structural seasonality of canopy height heterogeneity ($SS_{HH\mathrm{cv}}$), fractional cover (SS_{FC}), and structural complexity ($SS_{d\mathrm{D}}$). LCI and UCI describe the lower (5%) and upper (95%) interval coefficients, respectively.

Model	Regression	Latent variables	Estimate (LCI — UCI)	z-value (*p-value*)	Standardized latent	Standardized all
Height heterogeneity	Diversity	Taxonomic	1.00 (1.00 — 1.00)	—	0.33	0.96
	Diversity	Phylogenetic	0.47 (0.43 — 0.52)	21.16 ($p < 0.001$)	0.15	0.90
	Diversity	Functional	1.24 (1.17 — 1.31)	34.55 ($p < 0.001$)	0.40	1.02
	Variability	Taxonomic	1.00 (1.00 — 1.00)	—	0.49	0.96
	Variability	Phylogenetic	1.09 (1.04 — 1.17)	31.30 ($p < 0.001$)	0.54	1.02
	Variability	Functional	0.63 (0.37 — 0.85)	5.16 ($p < 0.001$)	0.31	0.64
	Structural stability	SS_{HHcv}	1.00 (1.00 — 1.00)	—	0.25	1.0
	NBE	NBE	1.00 (1.00 — 1.00)	—	0.48	1.0
Fractional cover	Diversity	Taxonomic	1.00 (1.00 — 1.00)	—	0.33	0.96
	Diversity	Phylogenetic	0.47 (0.42 — 0.52)	21.01 ($p < 0.001$)	0.15	0.90
	Diversity	Functional	1.23 (1.20 — 1.32)	33.75 ($p < 0.001$)	0.40	1.02
	Variability	Taxonomic	1.00 (1.00 — 1.00)	—	0.49	0.96
	Variability	Phylogenetic	1.10 (1.03 — 1.18)	16.64 ($p < 0.001$)	0.54	1.02
	Variability	Functional	0.63 (0.35 — 0.85)	5.04 ($p < 0.001$)	0.31	0.64
	Structural stability	SS_{FC}	1.00 (1.00 — 1.00)	—	0.33	1.0
	NBE	NBE	1.00 (1.00 — 1.00)	—	0.48	1.0
Structural complexity	Diversity	Taxonomic	1.00 (1.00 — 1.00)	—	0.33	0.96
	Diversity	Phylogenetic	0.47 (0.43 — 0.52)	21.07 ($p < 0.001$)	0.16	0.90
	Diversity	Functional	1.23 (1.16 — 1.31)	34.04 ($p < 0.001$)	0.40	1.02
	Variability	Taxonomic	1.00 (1.00 — 1.00)	—	0.49	0.96
	Variability	Phylogenetic	1.11 (1.04 — 1.21)	27.01 ($p < 0.001$)	0.54	1.02
	Variability	Functional	0.63 (0.36 — 0.85)	5.00 ($p < 0.001$)	0.31	0.63
	Structural stability	$SS_{d\mathrm{D}}$	1.00 (1.00 — 1.00)	—	0.30	1.00
	NBE	NBE	1.00 (1.00 — 1.00)	—	0.48	1.00

Table S6. Results from the structural regression models associated with the regression variables on models based on the forest structural seasonality of canopy height heterogeneity (SS_{HHcv}), fractional cover (SS_{FC}), and structural complexity ($SS_{d\mathrm{D}}$).

Model	Dependent	Independent	Estimate (LCI — UCI)	z-value (*p-value*)	Standardized latent	Standardized all
Height heterogeneity	Stability	Diversity	0.36 (0.25 — 0.46)	6.99 ($p < 0.001$)	0.47	0.47
		Variability	0.20 (0.12 — 0.29)	4.75 ($p < 0.001$)	0.41	0.41
	NBE	Diversity	-0.04 (-0.41 — 0.25)	-0.23 ($p = 0.82$)	-0.03	-0.03
		Variability	0.45 (0.19 — 0.76)	3.14 ($p < 0.01$)	0.45	0.45
		Stability	-0.65 (-1.18 — -0.05)	-2.29 ($p = 0.02$)	-0.33	-0.33
Fractional cover	Stability	Diversity	-0.02 (-0.19 — 0.13)	-0.24 ($p = 0.81$)	-0.02	0.02
		Variability	0.33 (0.17 — 0.49)	4.03 ($p < 0.001$)	0.50	0.50
	NBE	Diversity	-0.26 (-0.45 — -0.07)	-2.86 ($p < 0.01$)	-0.17	-0.17
		Variability	-0.03 (-0.27 — 0.12)	-0.33 ($p = 0.74$)	-0.03	-0.03
		Stability	1.05 (0.85 — 1.40)	7.49 ($p < 0.001$)	0.71	0.71
Structural stability	Stability	Diversity	-0.01 (-0.14 — 0.12)	-0.04 ($p = 0.96$)	-0.01	0.01
		Variability	0.30 (0.20 — 0.40)	5.91 ($p < 0.001$)	0.50	0.50
	NBE	Diversity	-0.28 (-0.45 — -0.12)	-3.39 ($p < 0.01$)	-0.19	-0.19
		Variability	-0.06 (-0.24 — 0.12)	-0.71 ($p = 0.48$)	-0.06	-0.06
		Stability	1.26 (1.02 — 1.50)	10.53 ($p < 0.001$)	0.77	0.77

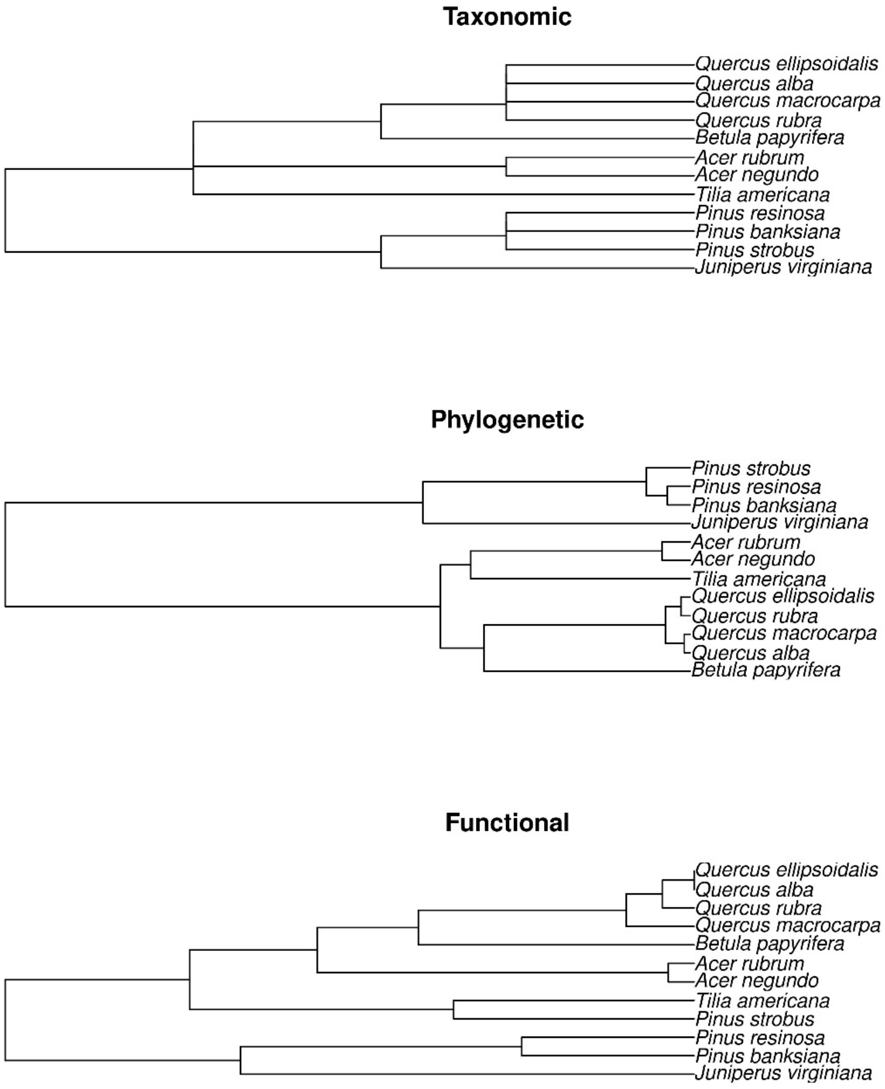

Figure S1. Taxonomic, phylogenetic, and functional trees of the twelve species present at the Forest and Biodiversity II experiment.

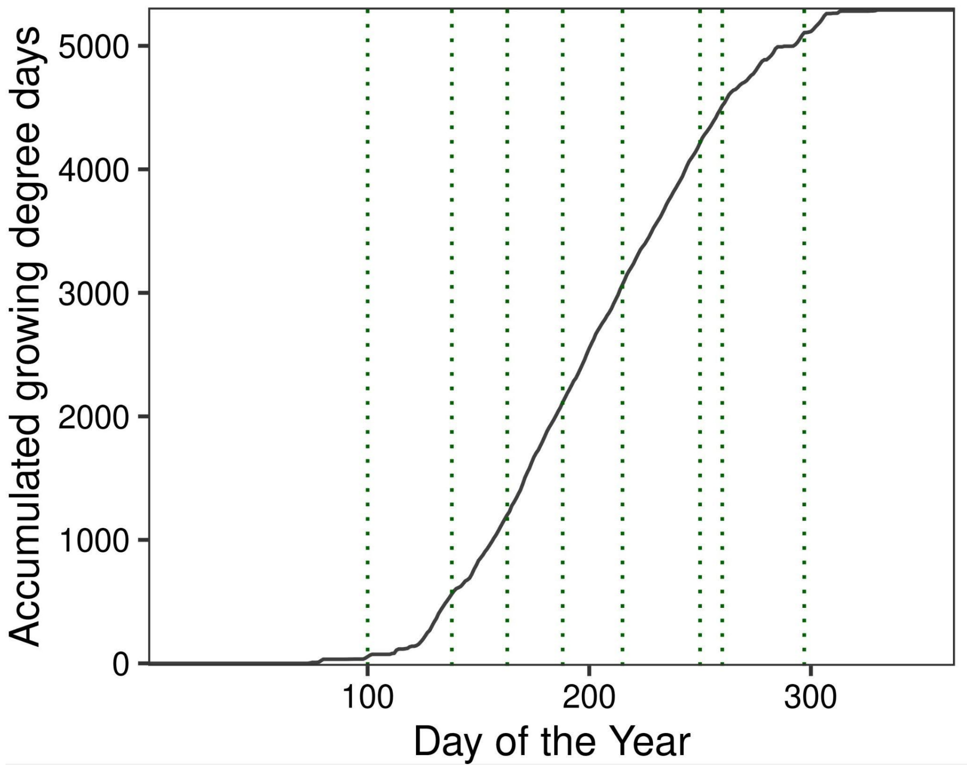

Figure S2. Accumulated growing degree days (base 15°C) for 2022 at the Cedar Creek Ecosystem Science Reserve (CCESR), Minnesota, USA. The estimated growing degree days were computed using meteorological measurements from Seeley (2023). Green dotted lines represent days of UAV surveys.

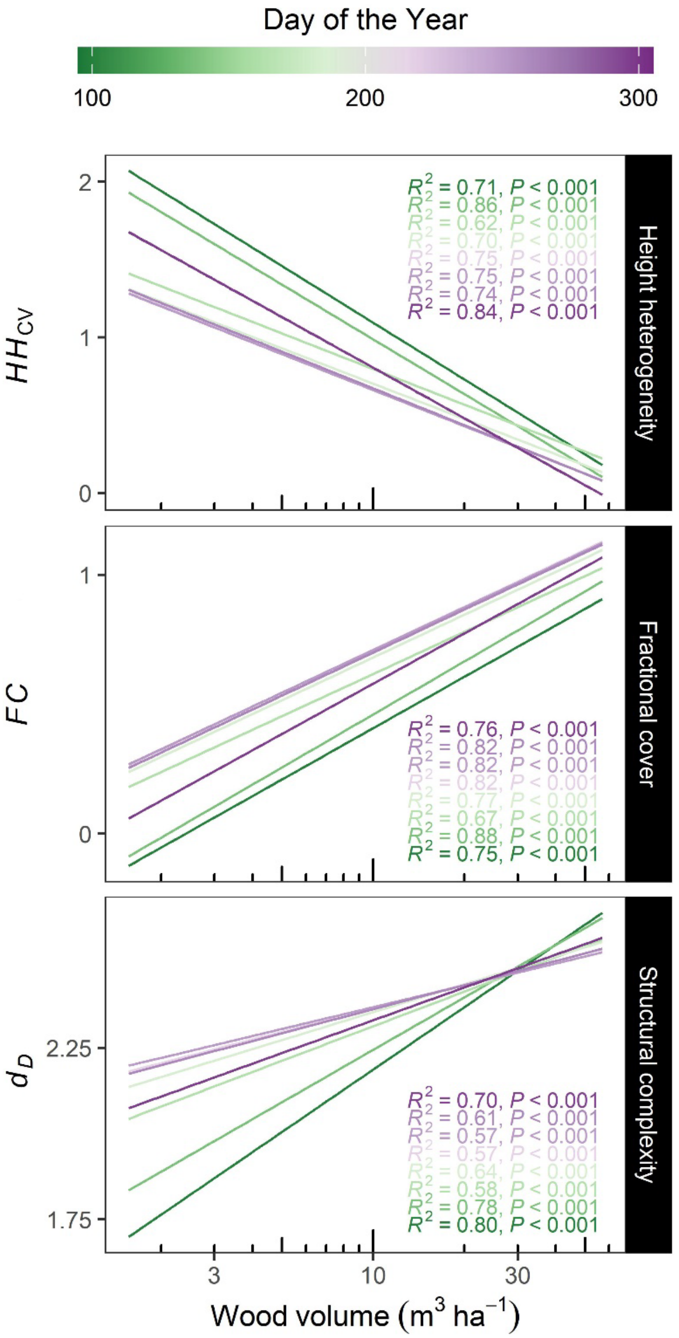

Figure S3. Relationships of plot wood volume with LiDAR-derived metrics at different observation periods through the growing season. HH_{CV} describes the coefficient of variation of canopy height heterogeneity, FC the fractional plant cover, and d_D the fractal dimension.

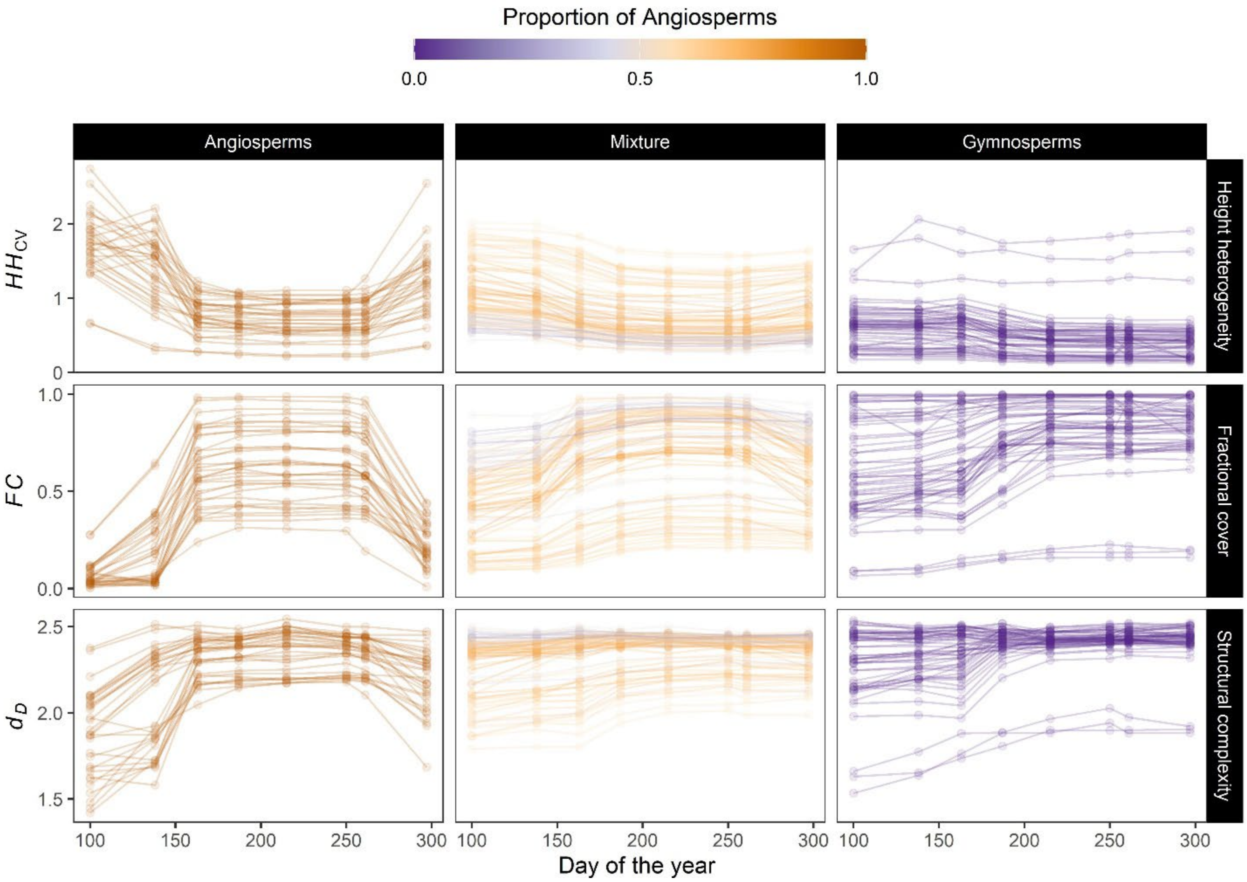

Figure S4. Temporal variation of height heterogeneity (the coefficient of variation of canopy height, HH_{CV}), fractional plant cover (FC), and structural complexity (fractal geometry, d_D) of plots composed of angiosperms, gymnosperms, and mixtures of angiosperms and gymnosperms. Each line represents a forest plot, and each point an observation. Colors represent the proportion of angiosperms that were planted in each plot.

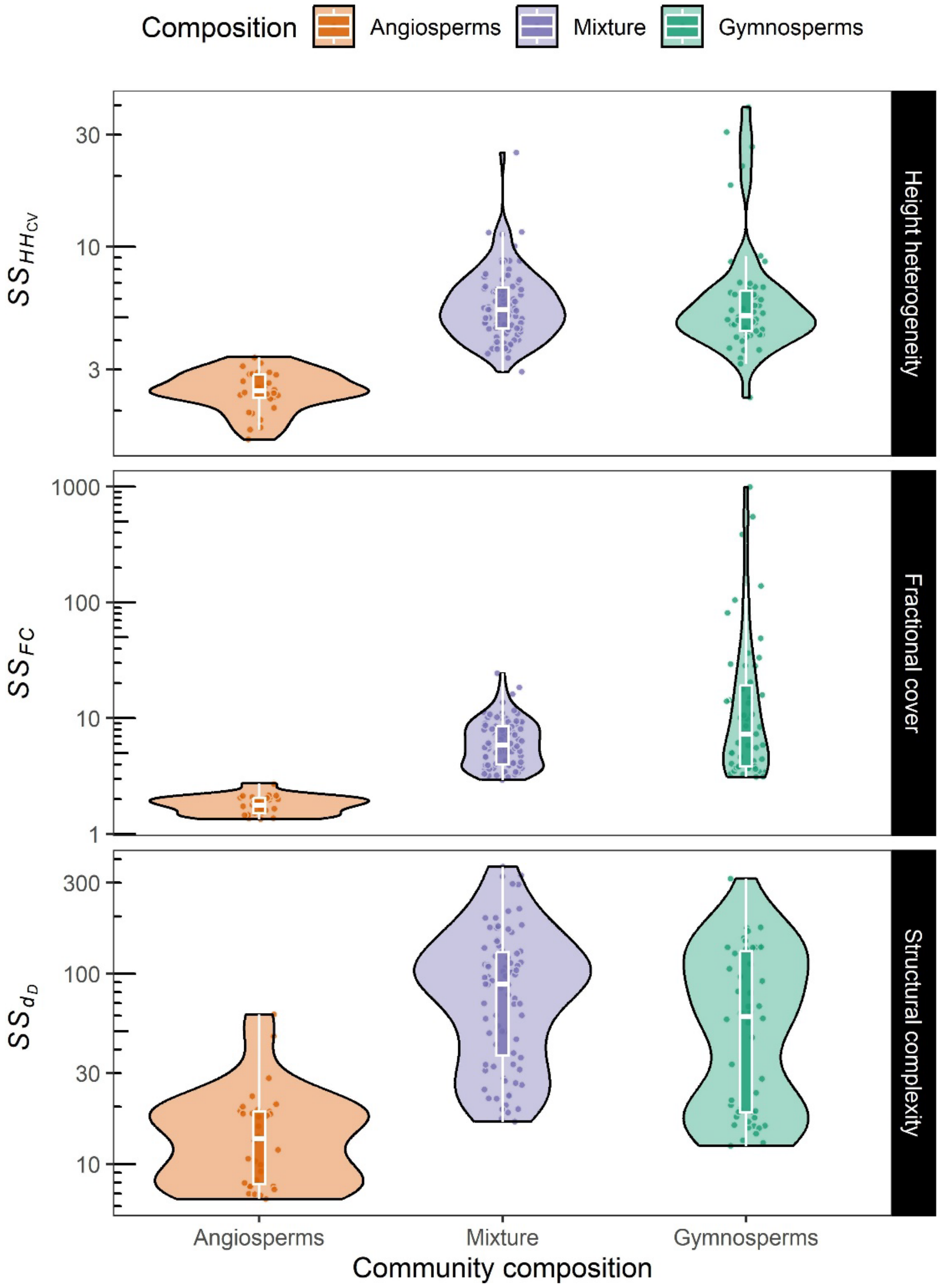

Figure S5. Comparison of seasonal structural stability (*SS*) of LiDAR-derived metrics among plots composed of angiosperms, gymnosperms, or mixtures (angiosperms and gymnosperms). HH_{CV} describes the coefficient of variation of canopy height, *FC* the fractional plant cover, and d_D the fractal dimension.

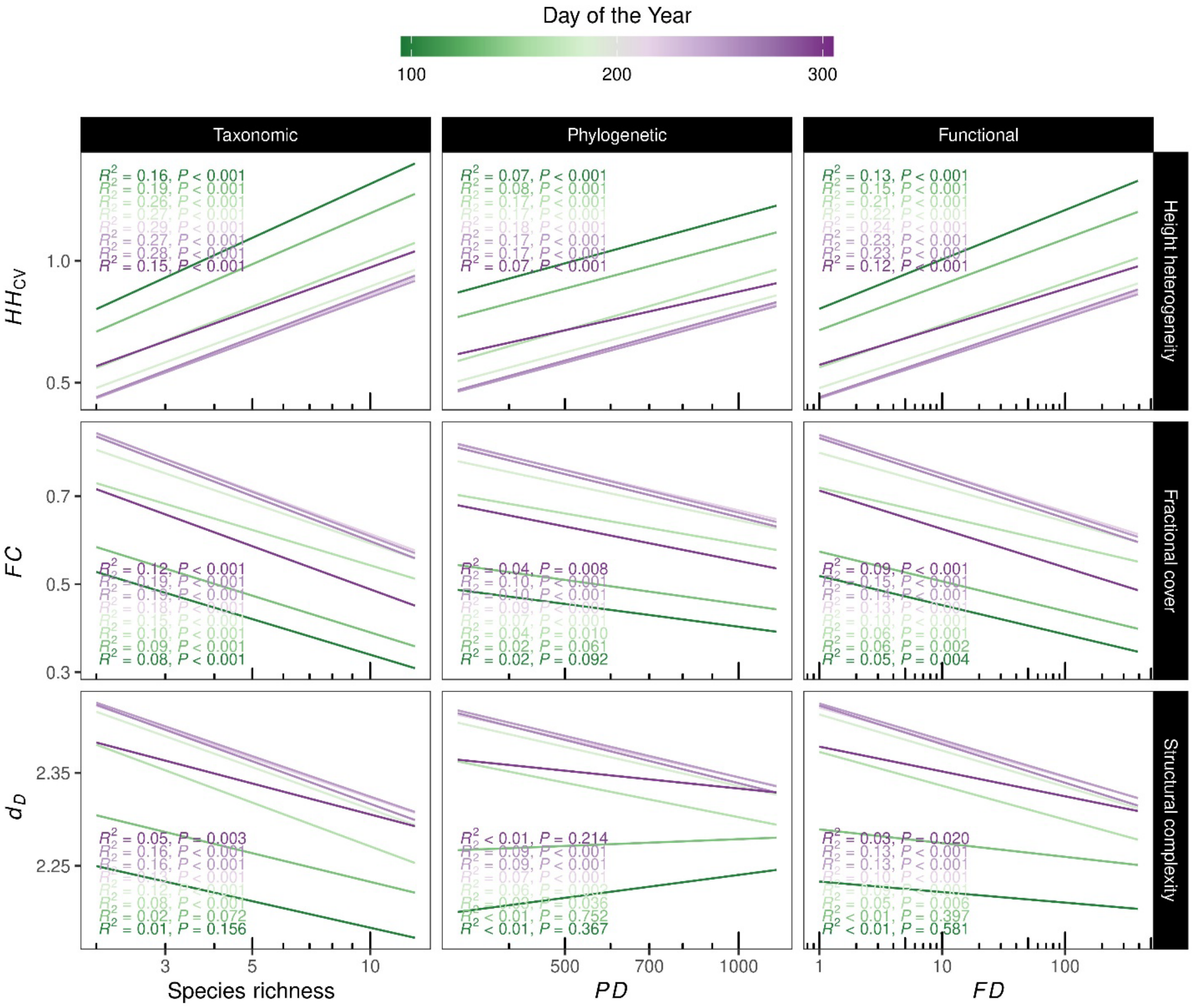

Figure S6. Association of multiple dimensions of diversity with LiDAR-derived metrics at different observation periods during the growing season. HH_{CV} describes the coefficient of variation in canopy height, *FC* the fractional plant cover, and d_{D} the fractal dimension.

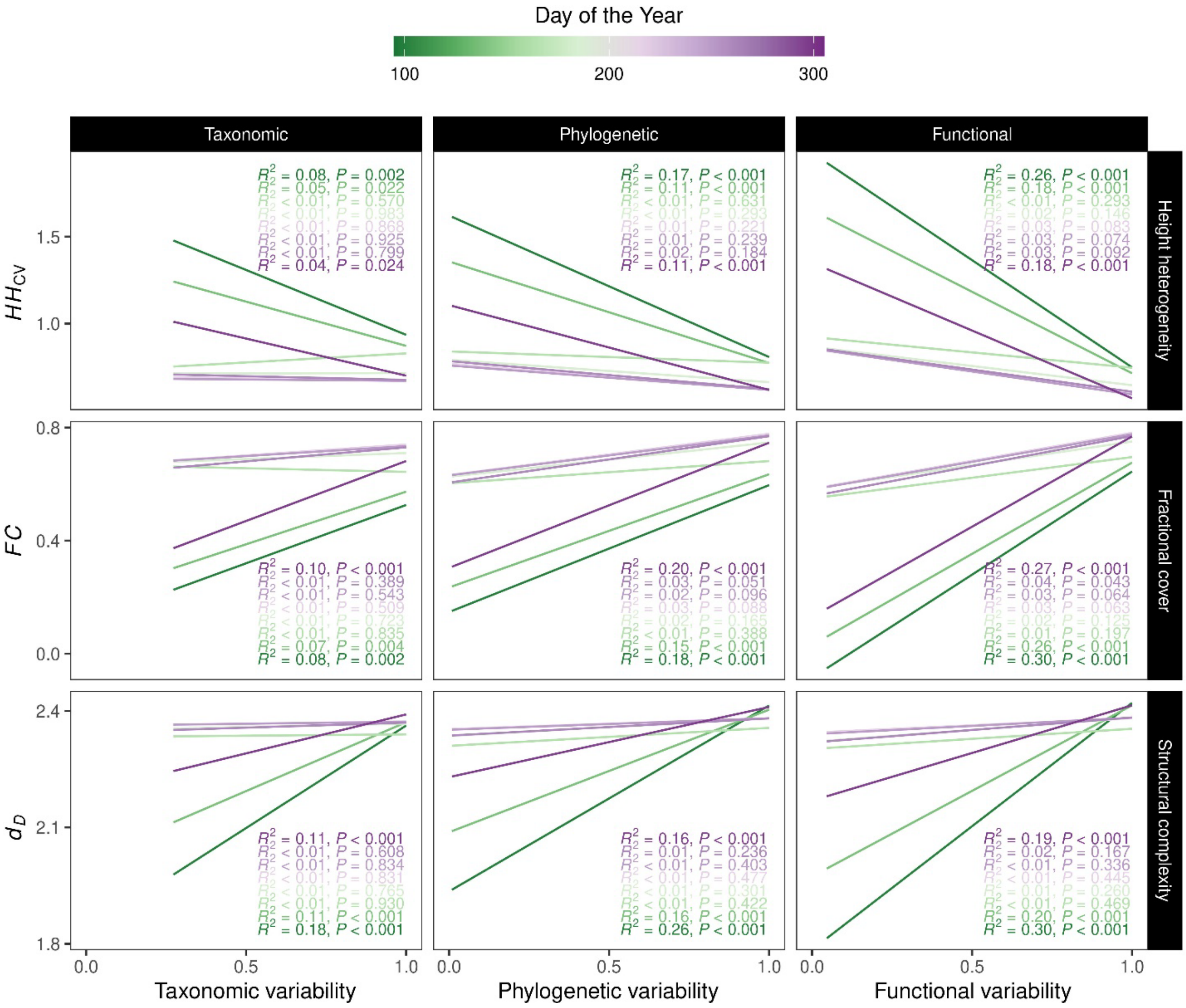

Figure S7. Association of multiple dimensions of variability with LiDAR-derived metrics at different observation periods during the growing season. HH_{CV} describes the coefficient of variation of canopy height, *FC* the fractional plant cover, and d_{D} the fractal dimension.

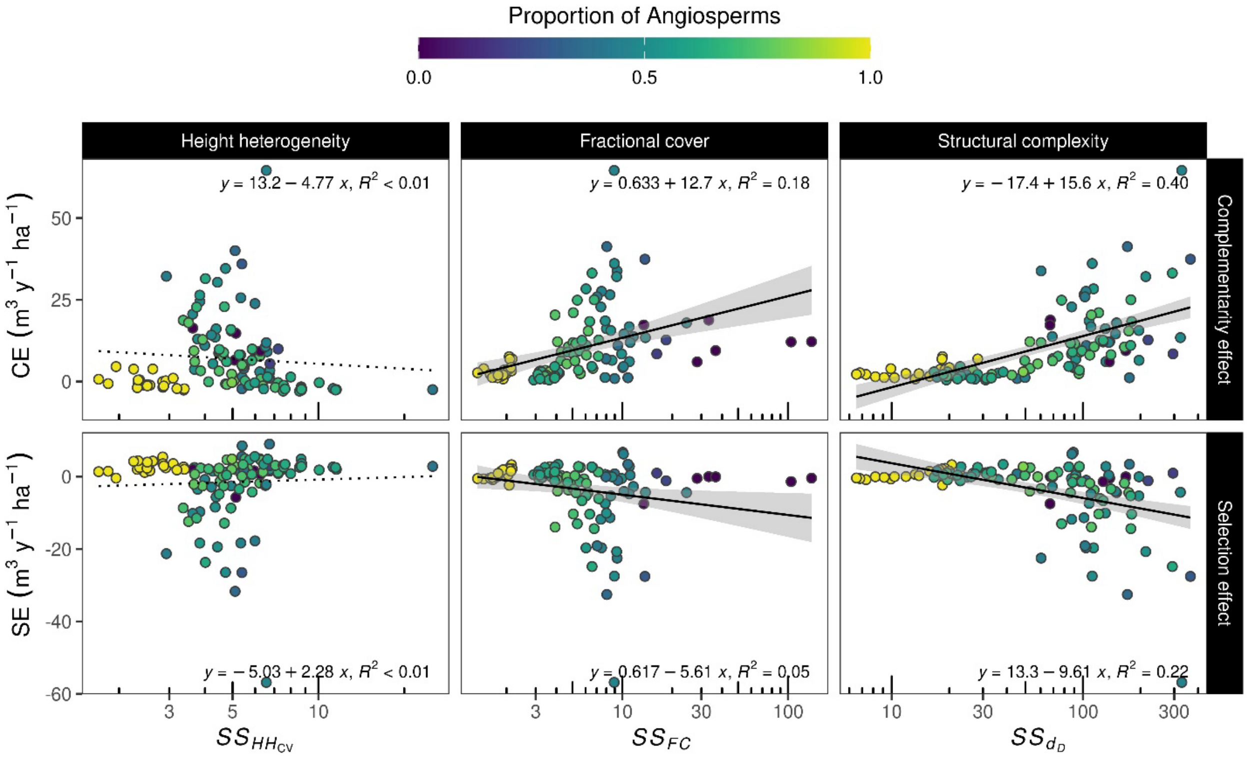

Figure S8. Relationship of seasonal structural stability (*SS*) of LiDAR-derived metrics with the complementarity (CE) and selection effect (SE) on annual wood productivity. HH_{CV} describes the coefficient of variation in canopy height, *FC* the fractional plant cover, and d_{D} the fractal dimension. Colors represent the proportion of angiosperms trees that were planted in each plot.

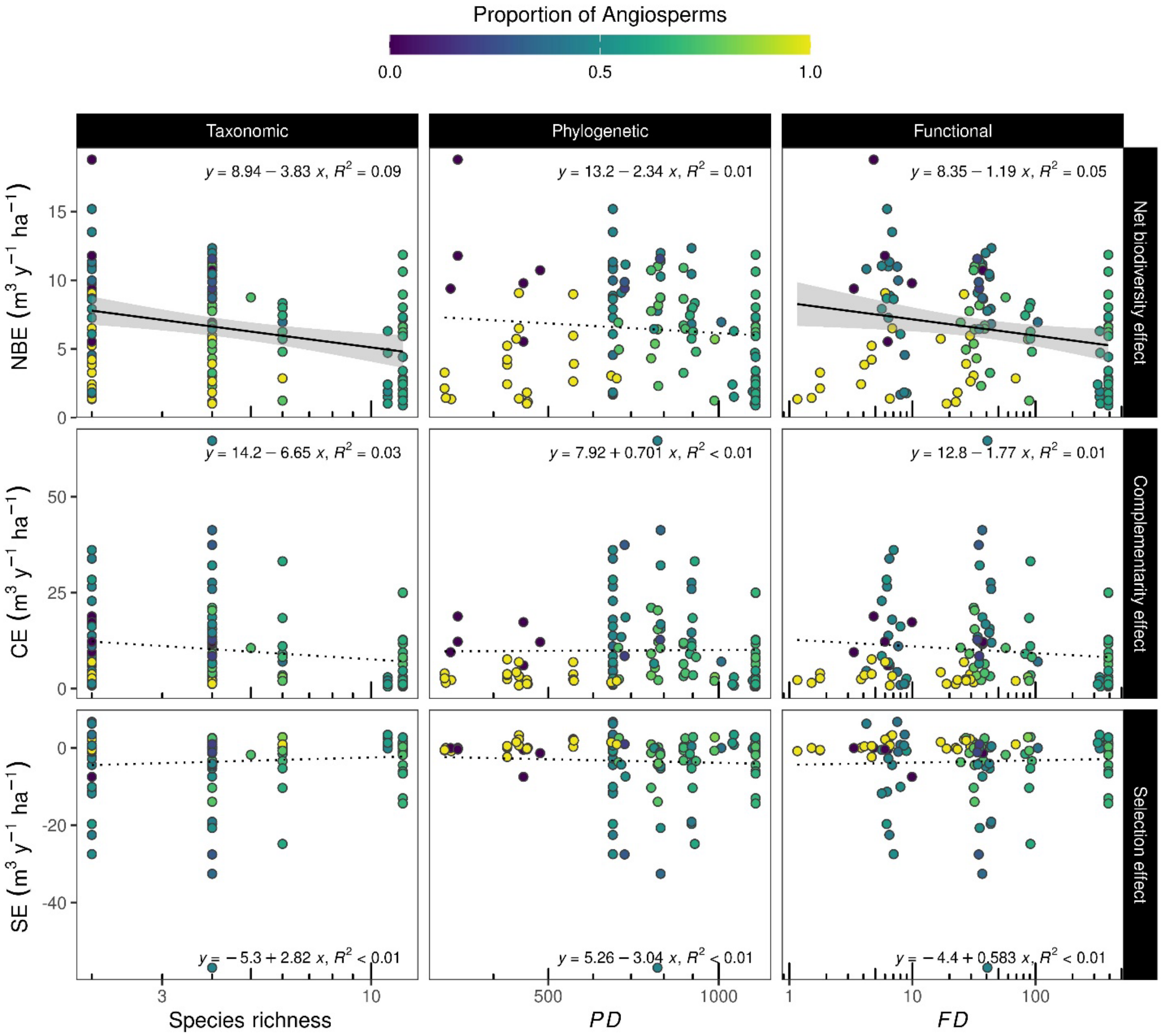

Figure S9. Association of multiple dimensions of diversity with net biodiversity (NBE), complementarity (CE), and selection (SE) effects on annual wood productivity.

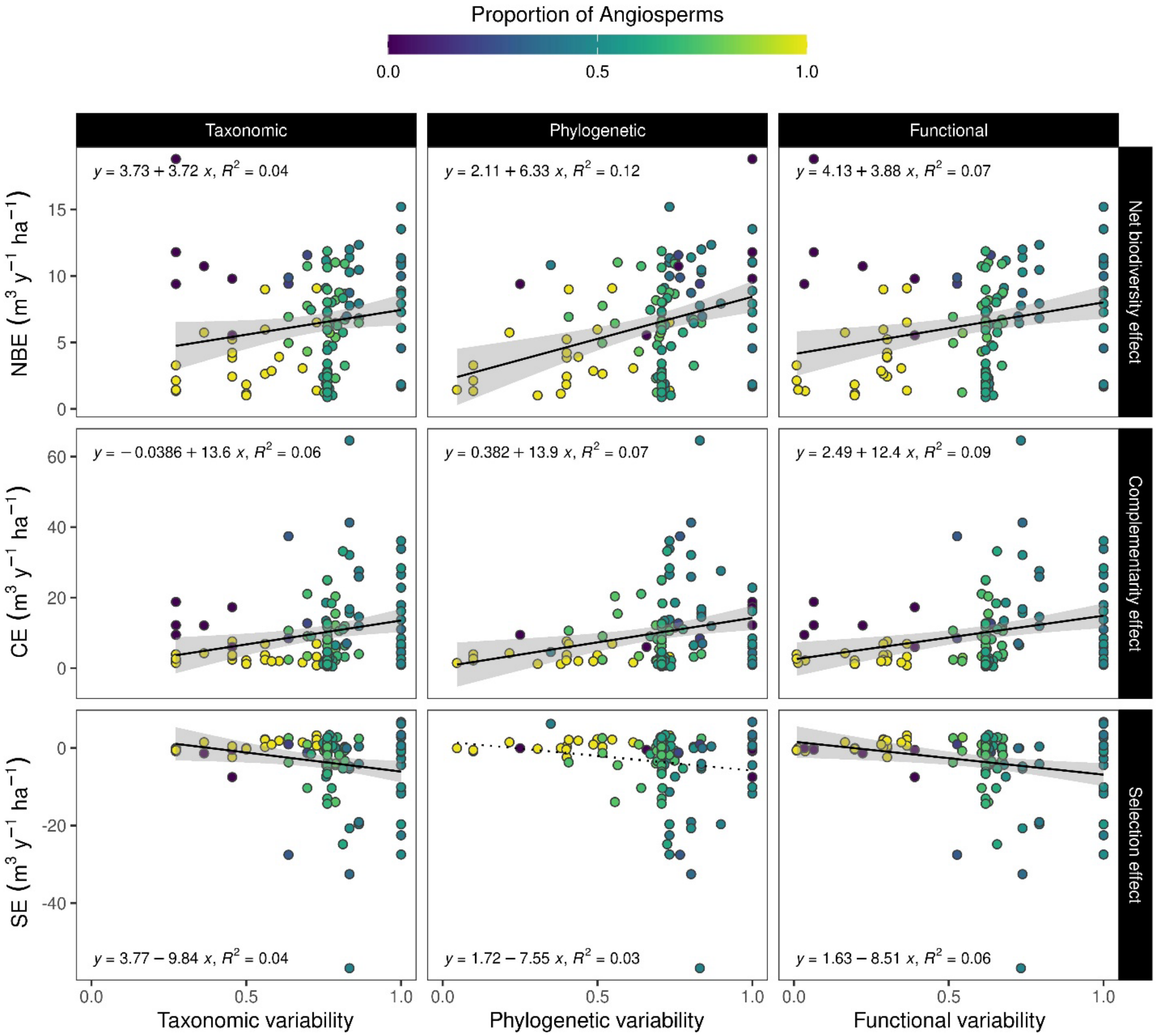

Figure S10. Relationship of multiple dimensions of species variability with net biodiversity (NBE), complementarity (CE), and selection (SE) effects on annual wood productivity.

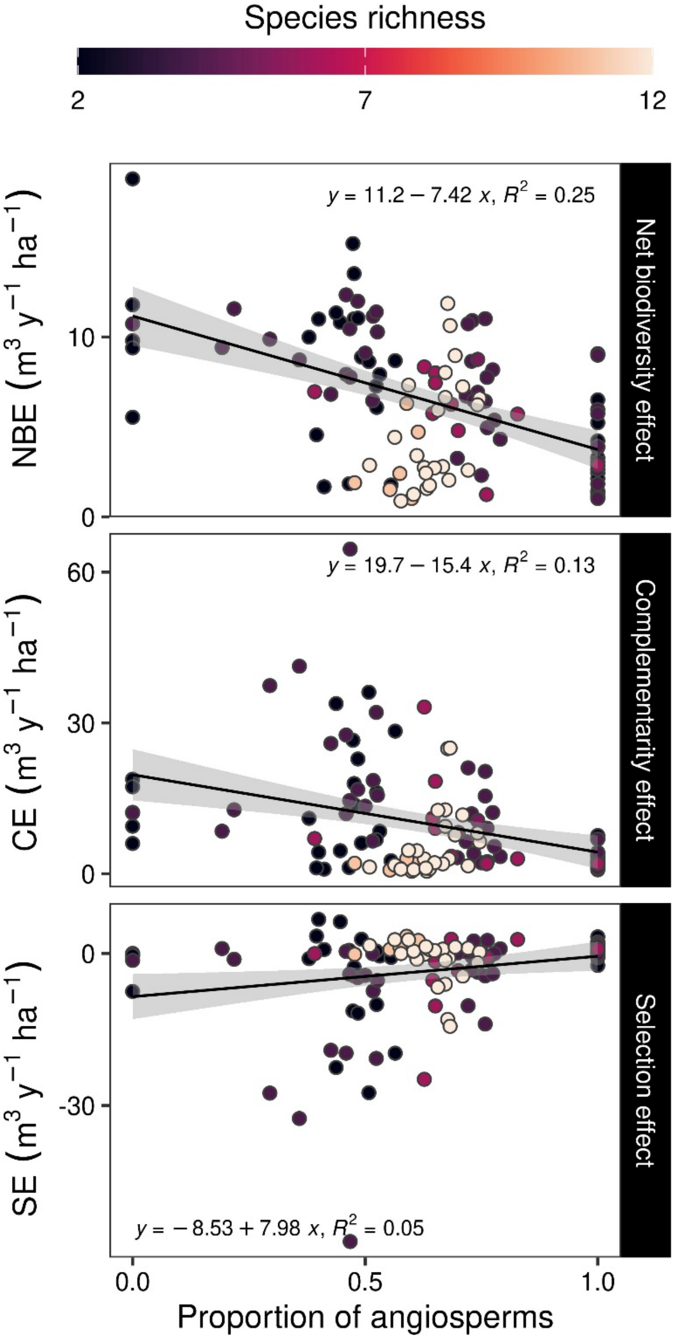

Figure S11. Association of the proportion of angiosperm trees with net biodiversity (NBE), complementarity (CE), and selection (SE) effects on annual wood productivity. Each point represents a plot.

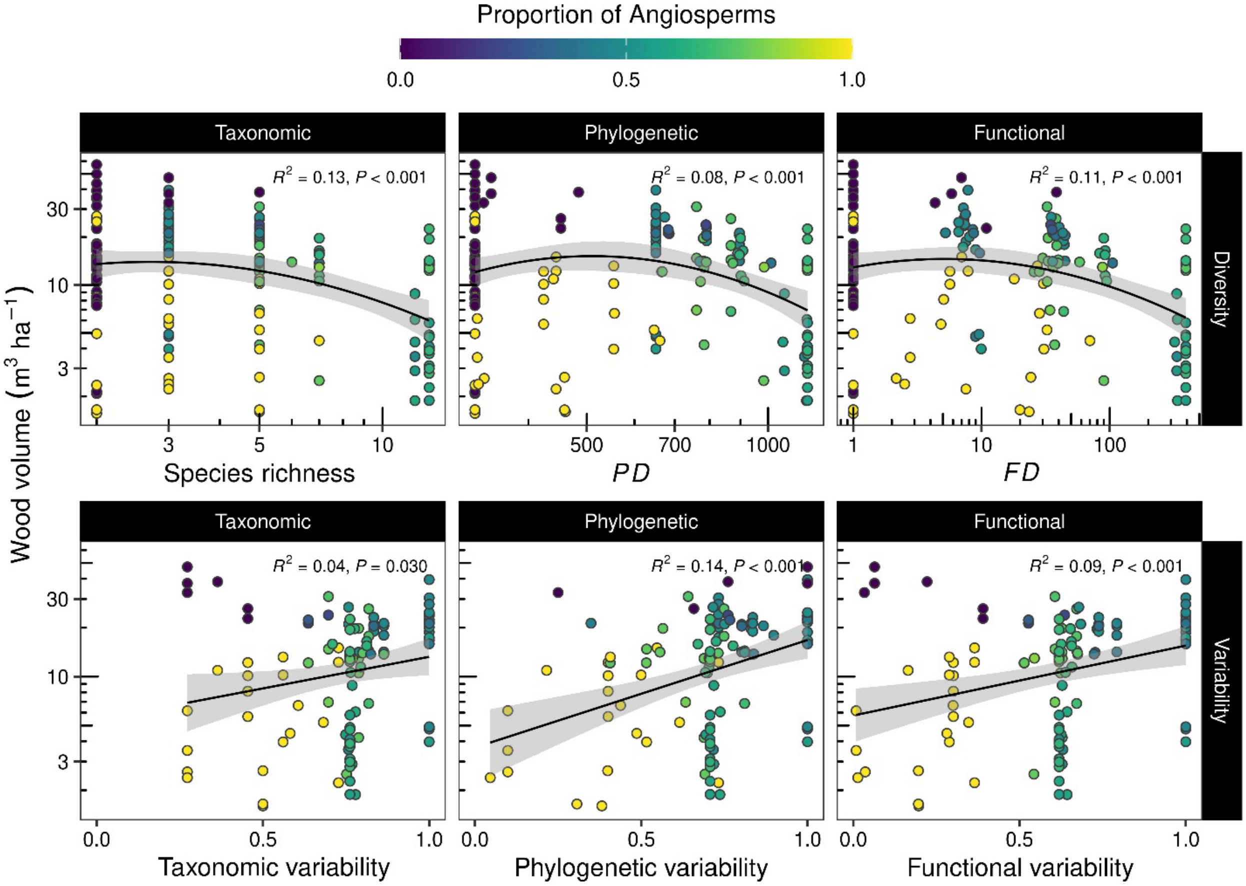

Figure 12. Relationships between multiple dimensions of diversity and variability and the wood volume on plots.

References

Grossman, J. J., J. Cavender-Bares, S. E. Hobbie, P. B. Reich, and R. A. Montgomery. 2017. Species richness and traits predict overyielding in stem growth in an early-successional tree diversity experiment. Ecology 98:2601–2614.

Isenburg, M. 2014. LAStools: Efficient LiDAR Processing Software. rapidlasso.

Kothari, S., R. A. Montgomery, and J. Cavender-Bares. 2021. Physiological responses to light explain competition and facilitation in a tree diversity experiment. Journal of Ecology 109:2000–2018.

Mattie, N. 2022, January 31. Strip Alignment and DTM generation through the estimation of the “median ground” terrain points. A methodology for DJI Zenmuse L1. https://www.linkedin.com/pulse/strip-alignment-dtm-generation-through-estimation-median-mattie.

Niinemets, Ü., and F. Valladares. 2006. Tolerance to shade, drought, and waterlogging of temperate Northern Hemisphere trees and shrubs. Ecological Monographs 76:521–547.

Seeley, M. 2023, November 15. Daily climate summary: Meterologic Measurements at Cedar Creek Natural History Area. Environmental Data Initiative.

Zanne, A. E., G. Lopez-Gonzalez, D. A. Coomes, J. Ilic, S. Jansen, S. L. Lewis, R. B. Miller, N. G. Swenson, M. C. Wiemann, and J. Chave. 2009, February 4. Data from: Towards a worldwide wood economics spectrum. Dryad.
